# Sustained YAP/TAZ activation promotes aberrant alveolar epithelial cell differentiation and drives persistent fibrotic remodeling

**DOI:** 10.1172/jci.insight.198113

**Published:** 2026-06-09

**Authors:** Isabella P. Gaona, A. Scott McCall, Natalie M. Geis, Arlo C. Colvard, Gianluca T. DiGiovanni, Taylor P. Sherrill, Ujjal K. Singha, David S. Nichols, Ana P. Serezani, Holly E. David, Jean-Philippe Cartailler, Shristi Shrestha, Sergey S. Gutor, Timothy S. Blackwell, Jonathan A. Kropski, Jason J. Gokey

**Affiliations:** 1 Division of Allergy, Pulmonary, and Critical Care Medicine, Department of Medicine, Vanderbilt University Medical Center, Nashville, Tennessee, USA.; 2 Department of Veterans Affairs Medical Center, Nashville, Tennessee, USA.; 3 Creative Data Solutions, Center for Stem Cell Biology, Vanderbilt University, Nashville, Tennessee, USA.; 4 Department of Internal Medicine, University of Michigan Medical School, Ann Arbor, Michigan, USA.; 5 Department of Cell and Developmental Biology, Vanderbilt University School of Medicine, Nashville, Tennessee, USA.

**Keywords:** Cell biology, Pulmonology, Adult stem cells, Fibrosis, Mouse models

## Abstract

YAP/TAZ signaling is required for initiation of lung alveolar repair, yet previous studies in idiopathic pulmonary fibrosis (IPF) predicted increased YAP/TAZ signaling in alveolar epithelial cells. We investigated whether persistent YAP/TAZ alveolar epithelial cell signaling contributes to failed epithelial repair and persistent fibrotic remodeling. In IPF lungs, we identified increased YAP^+^TAZ^+^ alveolar epithelial cells and increased transcriptional target expression. Pharmacological YAP/TAZ activation in human alveolar epithelial cell organoids and in murine AT2 cell organoids generated with genetic YAP/TAZ activation (YT^active^) (via deletion of Hippo kinases *Stk3* and *Stk4*) resulted in phenotype shifts into aberrant transitional and airway-like states. Bleomycin injury of YT^active^ mice resulted in persistent fibrotic remodeling at 28 and 56 days after bleomycin injury. Gene promoter activity associated with transitional cell markers (*Krt19*, *Hopx*, and *Runx2*) was increased in YT^active^ AT2 cells. Immunofluorescent staining showed a loss of AT2-associated Cebpa and increased *Krt19* in YT^active^ lineage-traced AT2 cells 28 days after injury. Inhibition of YAP/TAZ using verteporfin resulted in improved lung repair in YT^active^ mouse lungs, including restored Cebpa and decreased *Krt19^+^* transitional cells. These findings demonstrate that sustained YAP/TAZ activation drives abnormal alveolar repair and persistent fibrotic remodeling. Blocking aberrant persistent YAP/TAZ activity promotes adaptive repair and has potential as a therapeutic strategy for pulmonary fibrosis.

## Abstract 2

Persistent alveolar epithelial YAP/TAZ activity leads to abnormal transitional cell differentiation and failed alveolar repair that promotes development and progression of pulmonary fibrosis.

## Introduction

Idiopathic pulmonary fibrosis (IPF) is a chronic and progressive lung disease characterized by the replacement of functional alveoli with dense fibrotic scarring (1, 2). This fibrotic remodeling is associated with loss of respiratory function and is often lethal within 3–5 years of diagnosis. The only two FDA-approved treatments for IPF modestly slow loss of respiratory function, but do not stabilize disease or improve quality of life for IPF patients (3–5). The cause of IPF is unknown, but evidence from genetic studies and mouse models indicates that repetitive injury to — and failed repair of — the alveolar epithelium is a driving factor of disease progression that leads to activation of the fibroblast (6–10).

In the human and mouse lung alveolar epithelium, the alveolar type 2 (AT2) cells act as facultative progenitor cells during alveolar repair, proliferating to generate more AT2 cells and differentiating into alveolar type 1 (AT1) cells that lay in close proximity to the alveolar capillaries to facilitate gas exchange (11–14). During “failed” alveolar repair, aberrant progenitor AT2 populations can form “hyperplastic AT2” regions or differentiate into persisting alveolar transitional cell populations expressing markers of both AT1 and AT2 cells that do not adopt the long thin AT1 cell shape that is essential to facilitate gas exchange (15–18). These abnormal repair processes are believed to be an early driver of pulmonary fibrosis; thus, understanding the mechanisms that drive normal “adaptive” repair versus those that lead to fibrotic remodeling is essential to develop treatments to halt disease progression and/or restore lung function.

Several developmental pathways that are typically quiescent during lung homeostasis become transiently activated during normal repair; however, persistent activation of these pathways is predicted to lead to aberrant injury responses (15, 16, 19, 20). Recent work has implicated the Hippo-YAP/TAZ pathway as being involved in AT2 to AT1 differentiation during development and repair (21–23). The Hippo-YAP/TAZ pathway consists of the Hippo components mammalian serine-threonine kinases 1 and 2 (MST1 and MST2, in humans; Stk4 and Stk3, respectively, in mice), which phosphorylate Lats1 and Lats2, which in turn phosphorylate YAP and TAZ. When this phosphorylation cascade is active, YAP and TAZ are sequestered in the cytoplasm; however, in the absence of phosphorylation, YAP and/or TAZ translocate to the nucleus, where they interact with DNA binding partners (24–26) (classically, TEADs [refs. 27–29], Runx2 [refs. 30, 31], and Smads [refs. 32–34]) to direct transcription of target genes associated with proliferation and differentiation. Previous work from our group and others determined that YAP/TAZ activation is required for alveolar repair, as deletion of YAP/TAZ or just TAZ in AT2 cells prevents AT1 cell differentiation (35–38). Our previous work demonstrated that nuclear TAZ is expressed in nearly all AT1 cells, while nuclear YAP or TAZ is rarely detected in AT2 cells at homeostasis. During repair, YAP and TAZ are activated in AT2 cells, with peak activity at 7 and 14 days, respectively, and are downregulated to low activity by 21–28 days after bleomycin injury (35). These studies demonstrate that some degree of YAP/TAZ activity is essential early during the repair process and preemptive inhibition of YAP/TAZ would be maladaptive for lung repair. In contrast, single-cell RNA sequencing (scRNA-seq) and immunofluorescence data from our group and others have predicted that increased YAP/TAZ activity is associated with aberrant epithelial cells and fibroblast activation in the IPF lung (37, 39–45). Therefore, we hypothesized that aberrant persistent AT2 cell YAP/TAZ activation prevents normal alveolar epithelial repair, results in persistent transitional cells, and promotes fibrotic remodeling in the lung. In these studies, we sought to define the role of sustained YAP/TAZ activity in AT2 cells during lung injury and subsequent fibrotic remodeling and test whether interrupting this persistent epithelial YAP/TAZ activity would promote adaptive repair and block or delay IPF progression.

## Results

**Figure 1:**
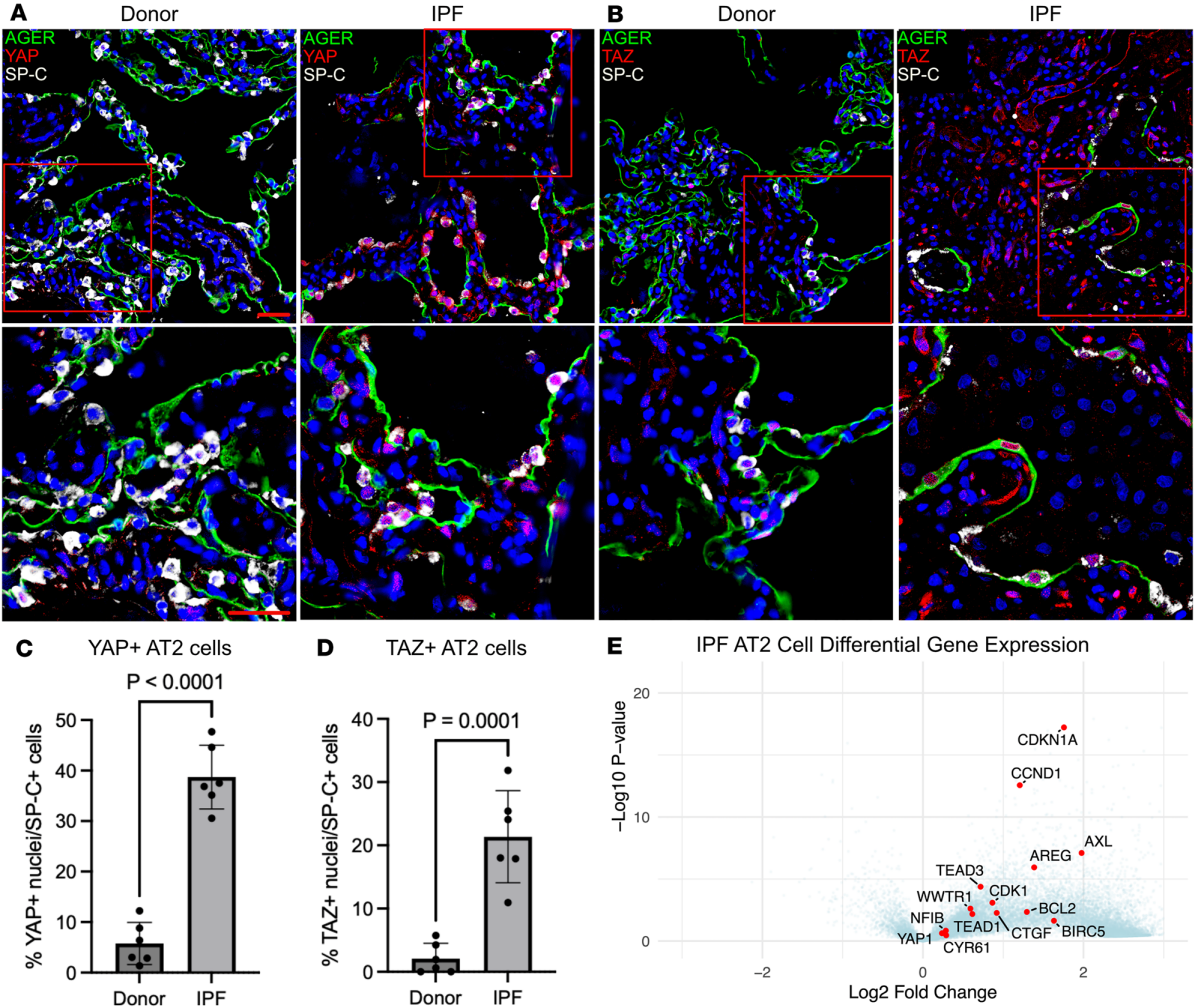
YAP and TAZ are active in the IPF epithelium. (**A** and **B**) Immunofluorescence analysis of YAP (red) (**A**) and TAZ (red) (**B**) in AGER^+^ (green) AT1 cells and SP-C^+^ (white) AT2 cells. Scale bars: 50 μm. (**C** and **D**) Quantification of YAP^+^ nuclei (**C**) and TAZ^+^ nuclei (**D**) in SP-C^+^ AT2 cells from donor and IPF lungs. *n* = 6 donors per group. Statistics determined using unpaired parametric *t* tests. (**E**) Differential gene expression analysis of IPF AT2 cells generated from previously published scRNA-seq analysis of IPF and donor lungs (GSE227136).

To determine whether YAP/TAZ is activated in the IPF lung epithelium, we used immunofluorescence staining to assess nuclear YAP or TAZ in cells positive for SP-C (marking either AT2 cells in healthy donors/IPF or hyperplastic/metaplastic alveolar epithelium in IPF) or for AGER (labeling AT1 in healthy donors/IPF or aberrant epithelium in IPF). Deidentified donor and IPF subject (*N* = 6 each) demographics are reported in Supplemental Table 1 (supplemental material available online with this article; https://doi.org/10.1172/jci.insight.198113DS1). Consistent with our previous findings (41), YAP was rarely detected in SP-C^+^ cells (5.8% ± 4.2%) of healthy donors; however, YAP was present in the nucleus of 38.7% (± 6.3%) of SP-C^+^ cells, including cells that were both SP-C^+^ and AGER^+^, in IPF lungs (Figure 1, A and C). TAZ was also rarely detected (2.1% ± 2.4%) in healthy donor SP-C^+^ AT2 cells; however, nuclear TAZ was present in 21.4% (± 7.3%) of SP-C^+^ and SP-C^+^AGER^+^ cells in IPF lungs (Figure 1, B and D). Fluorescence intensity of YAP and TAZ was quantified in Sp-C^+^DAPI^+^ nuclei, which demonstrated that both YAP and TAZ fluorescence intensity was increased in Sp-C^+^ nuclei in IPF lungs (Supplemental Figure 1). We then interrogated scRNA-seq data from our recently published work (46, 47), performing AT2 cell type–specific, subject-level pseudobulk differential expression analysis comparing IPF and control AT2 cells. Pseudobulk analysis was used to account for the variability across subjects and factor in the spectrum of YAP/TAZ activation across all AT2 cells. We found that *YAP* and *TAZ* (*WWTR1*), YAP/TAZ binding partners (*TEAD1/3*, *NFIB*), and YAP/TAZ targets and associated targets (CYR [ref. 48], CTGF, AXL [ref. 49], BIRC5, CCND1 [ref. 50], AREG [ref. 51], BCL2 [ref. 51], CDKN1A [ref. 52]) were increased in IPF AT2 cells compared with control donor AT2 cells (Figure 1E). CDK1, which phosphorylates YAP/TAZ during cell division to promote cancer development and metastasis (53), was also increased, although the role in this context is unknown. These findings support the concept that there is sustained activation of YAP/TAZ signaling in IPF alveolar epithelium.

**Figure 2:**
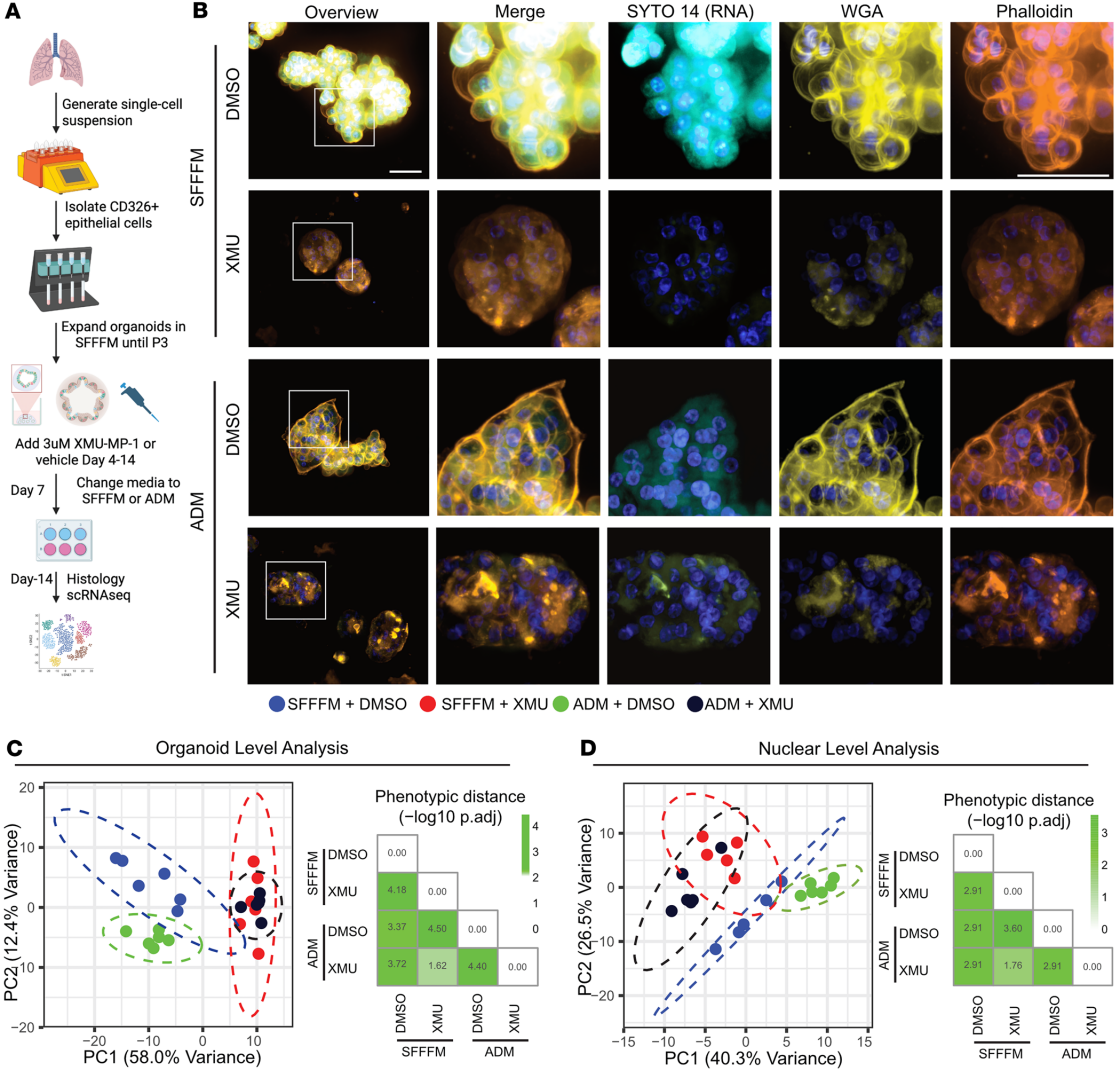
YAP/TAZ activation alters cell morphology in human organoids. (**A**) Schematic of human epithelial organoid generation, YAP/TAZ activation (XMU-MP-1), and collection strategy. (**B**) Cell and nucleus shape analysis using wheat germ agglutinin (WGA; yellow) and phalloidin (orange) with SYTO14 (cyan) in organoids treated with vehicle or XMU-MP-1 in either SFFFM or ADM. Scale bars: 50 μm. (**C**) Organoid morphologic analysis across phenotypic PCA space based on analysis of high-dimensional feature extraction and statistical analysis of phenotypic distance between groups. Each point represents wells as technical replicates. (**D**) Analysis of cell nuclei across phenotypic PCA space and phenotypic distance between groups. Each point represents wells as technical replicates. Two independent experiments were pooled for PCA analysis. *P* values were adjusted for multiple comparisons and derived from Hotelling’s *t* test in PCA space between groups.

**Figure 3:**
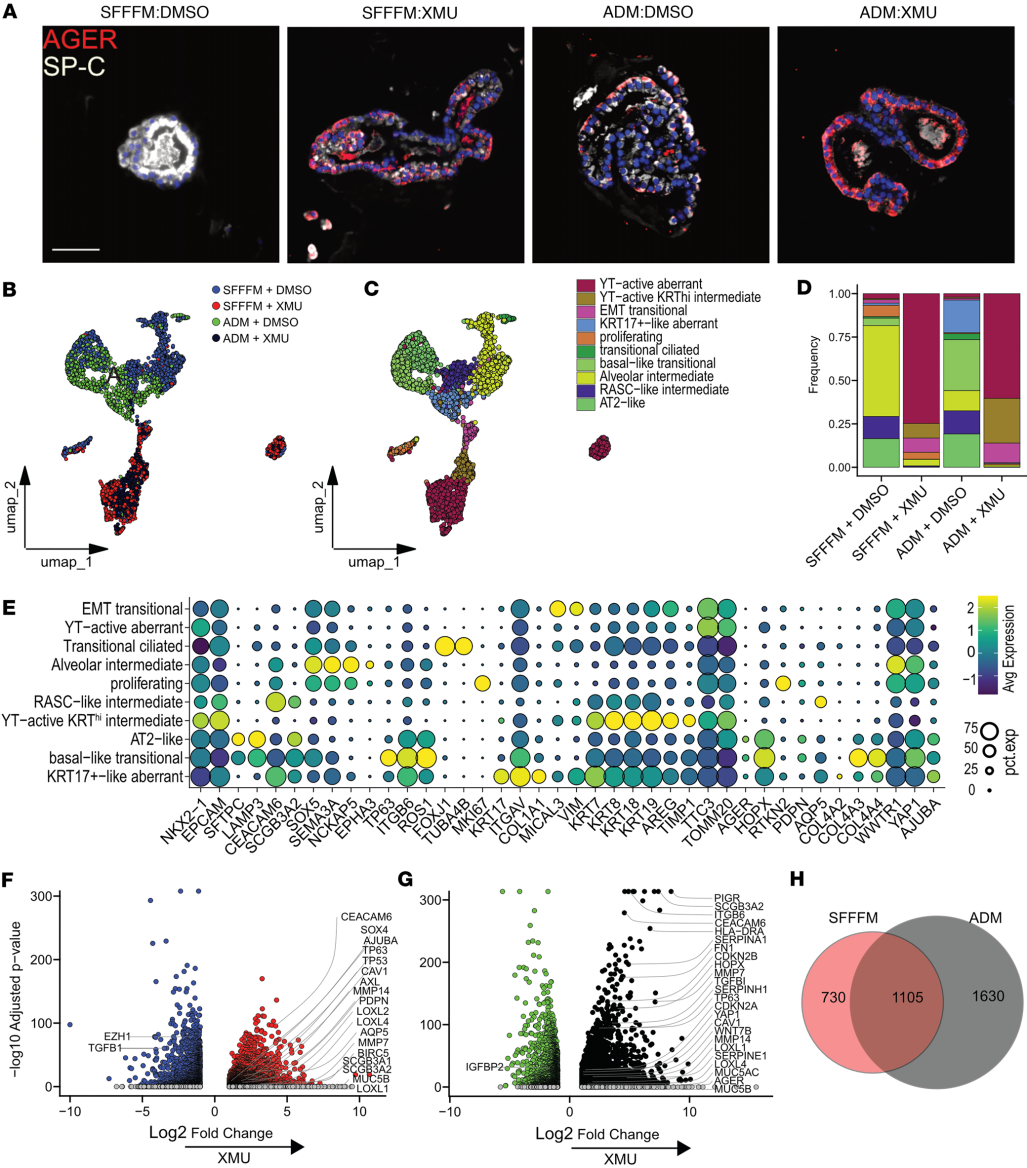
YAP/TAZ activation leads to aberrant epithelial cell populations in human alveolar organoids. (**A**) Immunofluorescence analysis of AGER^+^ cells and SP-C^+^ cells in organoids treated with 3 μM XMU-MP-1 or DMSO in expansion (SFFFM) and differentiation (ADM) media. Scale bar: 50 μm. (**B** and **C**) UMAP embedding of jointly analyzed scRNA-seq of YAP/TAZ–activated and DMSO control organoids annotated by treatment conditions under SFFFM or ADM (**B**) or cell type/state (**C**). Data from 2 independent experiments were pooled for analysis. (**D**) Stacked-bar quantification of percentages of identified cell populations. (**E**) Dot plot depicting marker gene expression in normal and aberrant cell populations. (**F**) Volcano plot of SFFFM versus SFFFM plus 3 μM XMU-MP-1 treatment with known YAP-mediated targets and genes associated with IPF within the alveolar epithelium noted. (**G**) Volcano plot of ADM versus ADM plus 3 μM XMU-MP-1 treatment with known IPF-associated and YAP-mediated targets within the alveolar epithelium noted. (**H**) Venn diagram noting the overlap of upregulated genes (*P*_adj_ < 0.05 and log_2_ fold change > 1) generated by 3 μM XMU-MP-1 between SFFFM and ADM.

To test the consequences of sustained YAP/TAZ signaling in human alveolar epithelial cells, CD326^+^ epithelial cells isolated from peripheral lung tissue obtained from declined lung donors were cultured in serum-free, feeder-free medium (SFFFM) as alveolar organoids (54). This approach allows the isolation of the heterogeneous populations, as recent work demonstrated that HTII-280 is not specific to AT2 cells, particularly in the IPF lung (55). Organoids were grown from day 1 to day 7 in SFFFM and then either continued in SFFFM until day 14 or switched to alveolar differentiation medium (ADM) from day 7 to day 14. Cells were treated with either vehicle or the YAP/TAZ–activating drug XMU-MP-1 (1 or 3 mM), a specific MST1/2 inhibitor (56), from culture day 4 to 14, during which we observed dramatic changes in organoid appearance (Figure 2, A and B, and Supplemental Figure 2A). RNA analysis by qPCR demonstrated that XMU-MP-1 treatment activated the YAP/TAZ target genes *AJUBA* and *CTGF* (Supplemental Figure 2B). We noted morphologic differences in culture and thus sought to characterize multiple subcellular components to quantify cellular morphologies. Whole-mount staining of RNA (SYTO14) and cytoskeletal component (wheat germ agglutinin and phalloidin) dyes followed by high-content imaging and analysis were used (57). We found that activation of YAP/TAZ with XMU-MP-1 resulted in marked and overriding morphologic changes of these human AT2 cell organoids whether cultured in SFFFM or commercial ADM, which drove unique morphologic appearance (ADM resulting in a splayed appearance) (Figure 2B and Supplemental Figure 2A). Analysis of vehicle- and XMU-treated organoids revealed cell morphology changes in YAP/TAZ–activated organoids as demonstrated by visualization of the variance in cell shape in phenotypic principal component analysis (PCA) space derived from all extracted morphologic features with substantial shifts between SFFFM, ADM, and XMU-treated organoids (58) (Figure 2C and Supplemental Figure 2, C and D). Likewise, analysis of cell nucleus morphology in phenotypic space incorporating RNA nuclear localization revealed considerably altered nuclear features in YAP/TAZ–activated organoids, which is consistent with prior YAP/TAZ activation phenotypes observed in mechanical situations (44, 59–61) (Figure 2D and Supplemental Figure 3). We then further characterized these organoids using immunofluorescent staining and scRNA-seq. Collected organoids were stained for AGER and SP-C, which demonstrated that YAP/TAZ activation with XMU increased the expression of AT1 cell–associated AGER in comparison with untreated organoids in SFFFM, suggesting increased differentiation toward AT1 or transitional cell populations (Figure 3A). Analysis of scRNA data indicated that while protein levels of the AT1 marker AGER were increased, sustained activation of YAP/TAZ signaling resulted in the aberrant differentiation of AT2 cells into several subpopulations, including a YAP/TAZ–active aberrant population expressing high levels of the mitochondrial marker *TOMM20* and a YAP/TAZ–active population expressing high levels of keratins (KRT^hi^) associated with aberrant transitional cell populations (Figure 3, B and C). These aberrant populations were present in XMU-MP-1–treated cultures regardless of SFFFM or ADM culture media or donor (Figure 3, D and E, and Supplemental Figure 4). Further supporting this concept, YAP/TAZ target genes (*AJUBA*, *AXL*, *BIRC5*) were increased in SFFFM conditions (Figure 3F), while AT1 cell markers *AGER*, *CAV1*, and *HOPX* were increased in ADM conditions (Figure 3G). However, aberrant airway markers including, *CEACAM6* and *P63* (basal/basaloid), *MUC5B* (goblet), *SCGB3A2* (distal secretory), as well as fibrotic associated genes *MMP7/14* and the lysyl oxidases *LOXL1* and *LOXL4* were increased in both media conditions (Figure 3H). These findings indicate that sustained activation of YAP/TAZ in human epithelial cells results in abnormal alveolar cell behavior and differentiation into aberrant cell populations, which supersedes signaling provided by media associated with either AT2 cell maintenance or components of alveolar differentiation.

**Figure 4:**
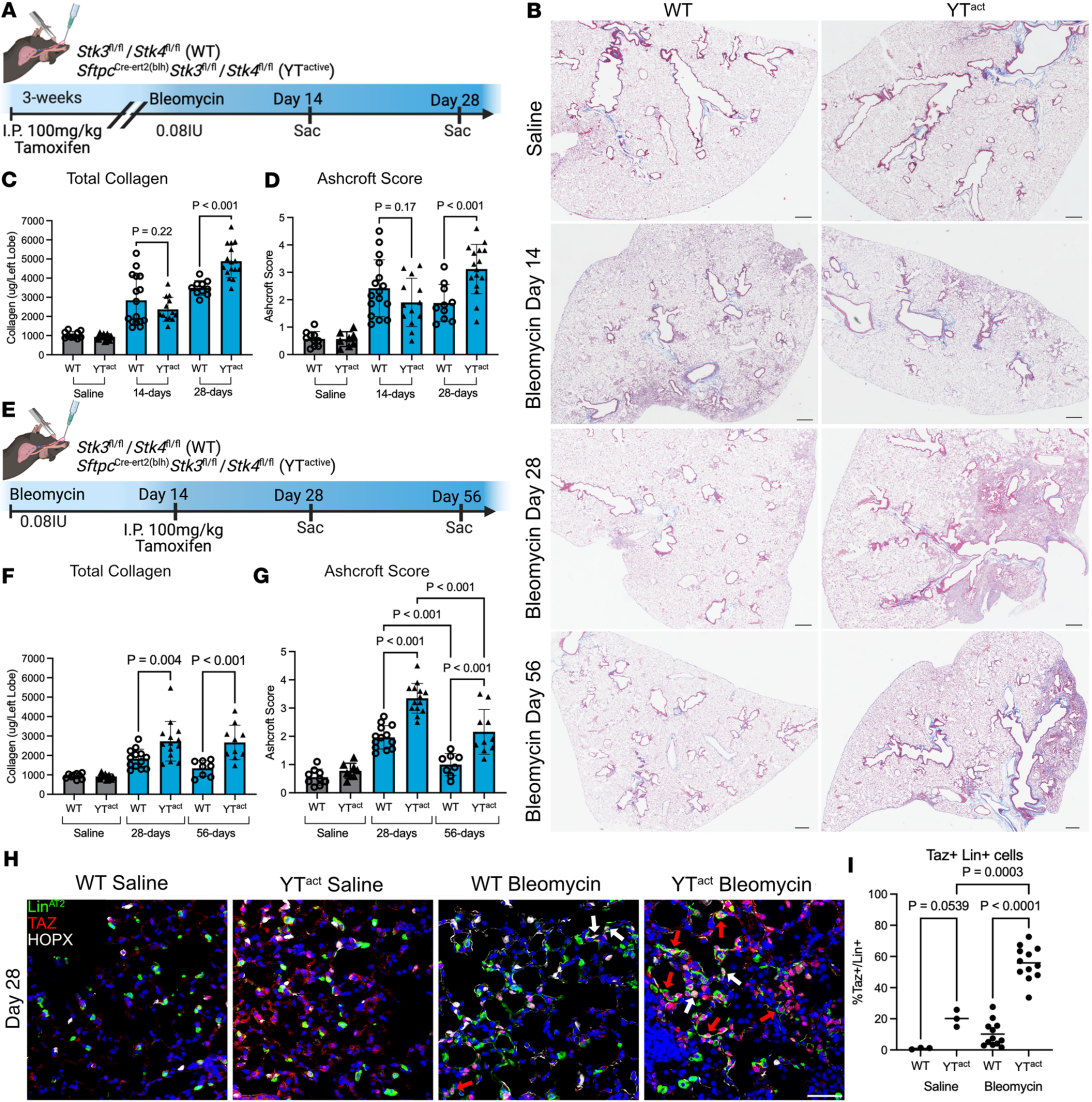
YT^active^ mice show increased and sustained fibrotic remodeling. (**A**) Timeline of mouse injury model with YAP/TAZ activated before bleomycin with lungs assessed at 14 and 28 days. (**B**) Masson’s trichrome staining of lung tissue sections from WT and YT^active^ mice at 14, 28, and 56 days after bleomycin or saline instillation. Scale bars: 100 μm. (**C** and **D**) Quantitative analysis of total collagen (**C**) and Ashcroft scoring of fibrosis (**D**) in WT and YT^active^ mice at 14 and 28 days after saline or bleomycin. Mice from 3 independent experiments were pooled in the following groups: WT saline (*n* = 10), YT^active^ saline (*n* = 8), day 14 WT bleomycin (*n* = 16), day 14 YT^active^ bleomycin (*n* = 13), day 28 WT bleomycin (*n* = 10), and day 28 YT^active^ (*n* = 16) bleomycin. (**E**) Timeline of mouse lung injury model with YAP/TAZ activated after bleomycin and collection at 28 and 56 days. (**F** and **G**) Quantitative analysis of total collagen (**F**) and Ashcroft scoring of fibrosis (**G**) in WT and YT^active^ mice at 28 and 56 days after saline or bleomycin. Mice across 3 independent experiments were grouped as WT saline (*n* = 9), YT^active^ saline (*n* = 8), day 28 WT bleomycin (*n* = 13), day 28 YT^active^ bleomycin (*n* = 14), day 56 WT bleomycin (*n* = 8), and day 56 YT^active^ bleomycin (*n* = 10). Ordinary 1-way ANOVA with Šidák’s multiple-comparison test with a single pooled variance was used to compare groups. (**H**) Immunofluorescence analysis of lineage-traced AT2 cells (green) expressing Hopx (white) and TAZ (red) at 28 days after injury. Red arrows indicate TAZ^+^ lineage-traced cells. White arrows indicate Hopx^+^TAZ^+^ lineage-traced cells. Scale bar: 50 μM. (**I**) Quantification of TAZ^+^ lineage-traced AT2 cells in WT and YT^active^ saline-treated mice (*N* = 3 mice each) and bleomycin-treated mice (*N* = 12 mice each) from 28 days after injury across both time courses.

To test whether this YAP/TAZ–driven aberrant differentiation impacts experimental lung fibrosis, we used a mouse model of conditional YAP/TAZ activation in AT2 cells. Hippo kinase–floxed mice (*Stk3^fl/fl^Stk4^fl/fl^*) were crossed with tamoxifen-inducible *SftpcCre^ert2tdTomato^* mice to generate *SftpcCre^ert2tdTomato^ Stk3^fl/fl^Stk4^fl/fl^*, hereafter referred to as YT^active^ mice. This model mimics the mechanism of XMU treatment (inhibition of MST1/2) by genetically deleting *Stk4* and *Stk3* (the mouse homologous genes). Cre^+^ YAP/TAZ–active mice and Cre^–^ littermate controls or *SftpcCre^ert2tdTomato^* wild-type (WT) mice were administered tamoxifen (100 mg/kg i.p.) 3 weeks before a single intratracheal dose of saline or 0.08 IU bleomycin. This tamoxifen delivery time point allows for washout to specifically target AT2 cells before lung injury. Lungs were collected at 14 days and 28 days after bleomycin for assessment of fibrosis (Figure 4, A and B). While total collagen (Figure 4C) and Ashcroft scoring (Figure 4D) were similar in WT and YT^active^ on day 14 after bleomycin, by day 28 YT^active^ mice had increased Ashcroft score and lung collagen content compared with WT mice. This indicated that sustained YAP/TAZ activation in AT2 cells drives persistent lung fibrosis and suggested the effects were mediated through regulation of the repair/resolution phase. To specifically test whether YAP/TAZ activation in AT2 cells during repair/resolution exacerbates fibrosis, we administered bleomycin to YT^active^ mice and littermate controls; then, on day 14 after bleomycin, mice were given tamoxifen to delete *Stk3/4* (thus activate YT) or corn oil (control) (Figure 4E). This tamoxifen delivery time point targets all cells expressing *Sftpc* at 14 days during the repair process and may include cells not targeted in the earlier time course such as alveolar transitional or distal airway populations. YT^active^ mice had increased collagen content (Figure 4F) and worsened Ashcroft score (Figure 4G) at both day 28 and day 56 after bleomycin. Since epithelial deletion of YAP/TAZ has previously been shown to regulate the lung immune response, bronchoalveolar lavage fluid was collected from saline- and bleomycin-injured mice. Neither total immune cell or Diff-Quik analysis of recruited immune cell populations revealed differences between WT and YT^active^ mice at the time points assessed (Supplemental Figure 5, A and B). YAP/TAZ activation by *Stk3/4* deletion was assessed by immunofluorescence analysis of TAZ in lineage-traced AT2 cells as well as in Hopx^+^ cells to assess TAZ in AT1 cells (Figure 4H). Quantification demonstrated that the majority of Hopx^+^ cells expressed TAZ (Supplemental Figure 5C), while TAZ was rarely detected in WT mouse lineage-traced AT2 cells (Figure 4H). In contrast, TAZ^+^ lineage-traced cells were significantly increased in YT^active^ mice injured with bleomycin, 28 days after injury (Figure 4I). In WT bleomycin-injured mouse lungs, most of the TAZ^+^ lineage-traced cells that were present were also Hopx^+^, suggesting differentiation toward AT1 cells (Supplemental Figure 5D). However, in YT^active^ lungs, a lower percentage of TAZ^+^ lineage-traced cells were Hopx^+^, suggesting that sustained TAZ activity is not sufficient to drive AT2 to AT1 cell differentiation. Together, these results indicated that persistent activation of YT in AT2 cells prevented resolution of lung injury and resulted in persistent lung fibrosis.

**Figure 5:**
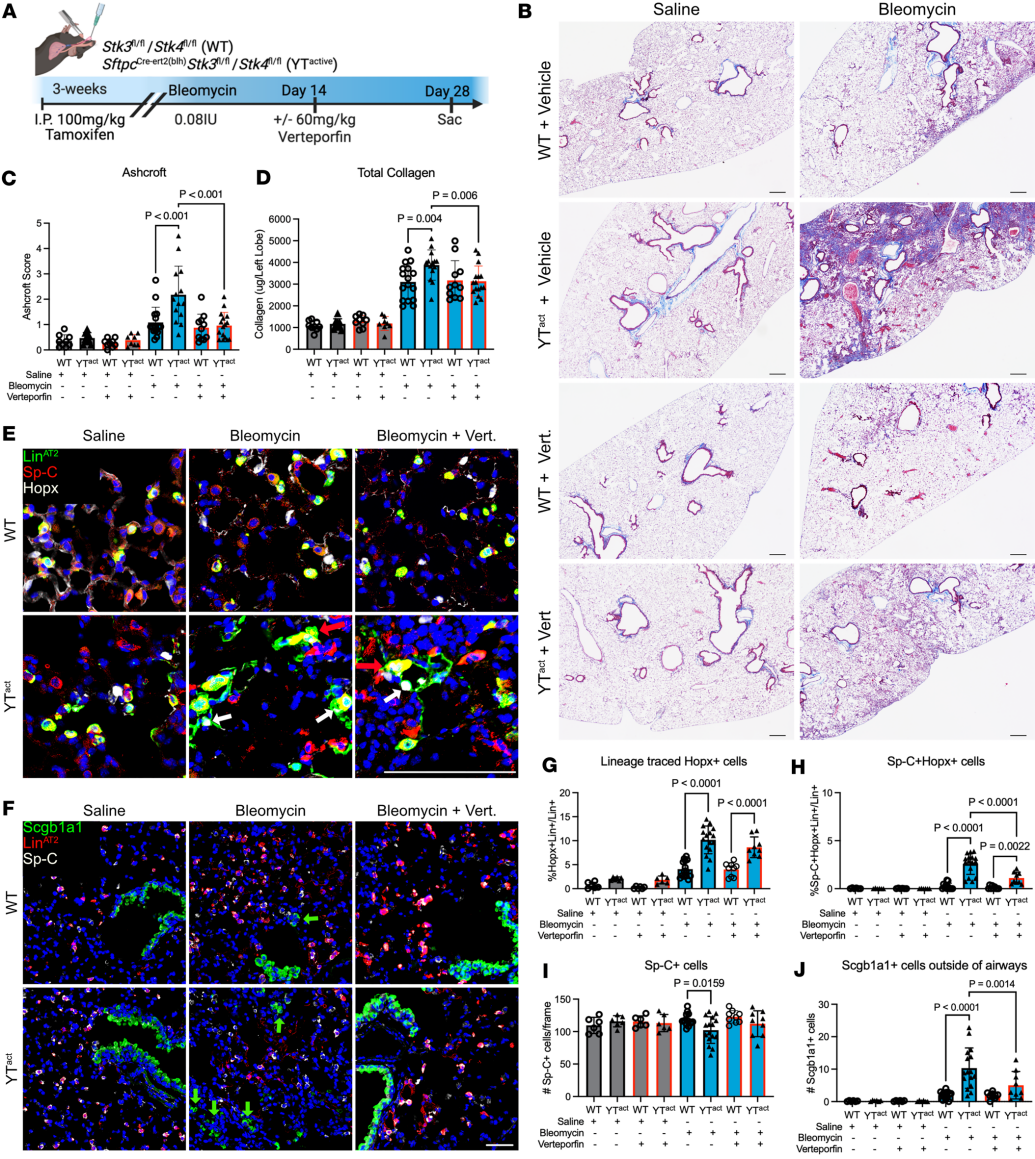
The YAP/TAZ inhibitor verteporfin partially rescues fibrotic phenotype in YT^active^ mice after bleomycin injury. (**A**) Timeline of YT^active^ mouse bleomycin injury model with YAP/TAZ inhibition with verteporfin (60 mg/kg) at 14 days after injury. (**B**) Masson’s trichrome staining of WT and YT^active^ mice with and without verteporfin following saline or bleomycin. Scale bars: 100 μm. (**C** and **D**) Ashcroft scoring of fibrosis (**C**) and total collagen analysis (**D**) of WT and YT^active^ mice with and without verteporfin following saline or bleomycin. Mice from 3 independent experiments were pooled in the following groups: WT saline (*n* = 9), YT^active^ saline (*n* = 9), WT saline/verteporfin (*n* = 9), YT^active^ saline/verteporfin (*n* = 8), WT bleomycin (*n* = 16), YT^active^ bleomycin (*n* = 14), WT bleomycin/verteporfin (*n* = 11), and YT^active^ bleomycin/verteporfin (*n* = 16). (**E**) Immunofluorescence analysis of Sp-C^+^ (red), Hopx^+^ (white), and lineage-traced AT2 (green) cells. White arrows indicate Hopx^+^ lineage-traced cells. Red arrows indicate Sp-C^+^Hopx^+^ lineage-traced cells. Scale bar: 50 μm. (**G**, **H**, and **I**) Quantification of lineage-traced AT2 cells expressing Hopx (**G**), Sp-C^+^Hopx^+^ cells (**H**), and total Sp-C^+^ cells (**I**) per frame. (**F**) Scgb1a1^+^ cells (green), AT2 lineage-labeled cells (red), and Sp-C^+^ cells (white) in WT and YT^active^ mice given saline, bleomycin, or bleomycin and verteporfin. Green arrows indicate Scgb1a1^+^ cells outside of visible airways. Scale bar: 50 μm. (**J**) Quantification of Scgb1a1^+^ cells outside of airways in the alveolar region. Mice from 3 independent experiments were grouped as WT and YT^active^ saline (*n* = 6), WT and YT^active^ saline/verteporfin (*n* = 6), WT bleomycin (*n* = 14), YT^active^ bleomycin (*n* = 16), WT bleomycin/verteporfin (*n* = 9), and YT^active^ bleomycin/verteporfin (*n* = 9). To determine significance, an ordinary 1-way ANOVA with Šidák’s multiple-comparison test with a single pooled variance was used.

Next, to determine whether intervention with a pharmacological YAP/TAZ inhibitor could restore alveolar repair/regeneration, tamoxifen-treated YT^active^ and WT mice were challenged with intratracheal bleomycin (3 weeks after tamoxifen, similar to Figure 4A) and injected with verteporfin (60 mg/kg i.p.), a YAP/TAZ–TEAD inhibitor that prevents YAP/TAZ nuclear localization and enhances degradation (48), 14 days after bleomycin-induced lung injury (Figure 5A). Consistent with the above findings, YT^active^ mice had increased collagen content and worsened lung injury scores at 28 days after injury (Figure 5, B–D). While verteporfin did not impact fibrosis in WT mice, verteporfin treatment of YT^active^ mice led to improved Ashcroft scores and lower total collagen content as measured by total Sircol in comparison with vehicle-treated YT^active^ mice at 28 days after injury (Figure 5, B–D). Immunofluorescence analysis of WT and YT^active^ mouse lungs treated with saline or bleomycin showed increased Hopx^+^ AT1-like cells derived from lineage-labeled AT2 cells, as well as increased Sp-C^+^Hopx^+^ transitional cells, in YT^active^ bleomycin-treated lungs (Figure 5, E–G). Verteporfin treatment did not significantly impact the number of lineage-traced Hopx^+^ cells; however, the number of transitional Sp-C^+^Hopx^+^ cells was reduced, suggesting resolution of the transitional phenotype. YT^active^ bleomycin-treated lungs had decreased overall Sp-C^+^ AT2 cells and increased Hopx^+^ AT1-like cells, which were not significantly affected by verteporfin treatment (Figure 5I and Supplemental Figure 6A). Beyond a role in AT2 to AT1 cell differentiation, YAP and TAZ are also known to regulate cell proliferation. Immunofluorescence analysis of Ki67^+^ proliferating cells found that YT^active^ bleomycin-injured mouse lungs had increased proliferating lineage-traced cells compared with WT bleomycin-injured lungs (Supplemental Figure 6B). The number of proliferating lineage-traced cells was reduced, although not significantly, by verteporfin treatment 28 days after injury (Supplemental Figure 6C). We observed that during single-dose bleomycin treatment, a small number of Scgb1a1^+^ airway cells (2.0% ± 1.1%) migrated into the distal lung to potentially participate in repair. Scgb1a1^+^ cells were increased (10.3% ± 6.2%) in the distal lung parenchyma in YT^active^ bleomycin-injured lungs in comparison with littermates, while verteporfin treatment led to reduced parenchymal Scgb1a1^+^ cells (5.0% ± 4.2%) in comparison with vehicle-treated YT^active^ mice (Figure 5, H and J). These findings indicate that verteporfin treatment after onset of fibrotic remodeling improved lung regeneration by antagonizing the sustained YAP/TAZ activity in AT2 cells.

**Figure 6:**
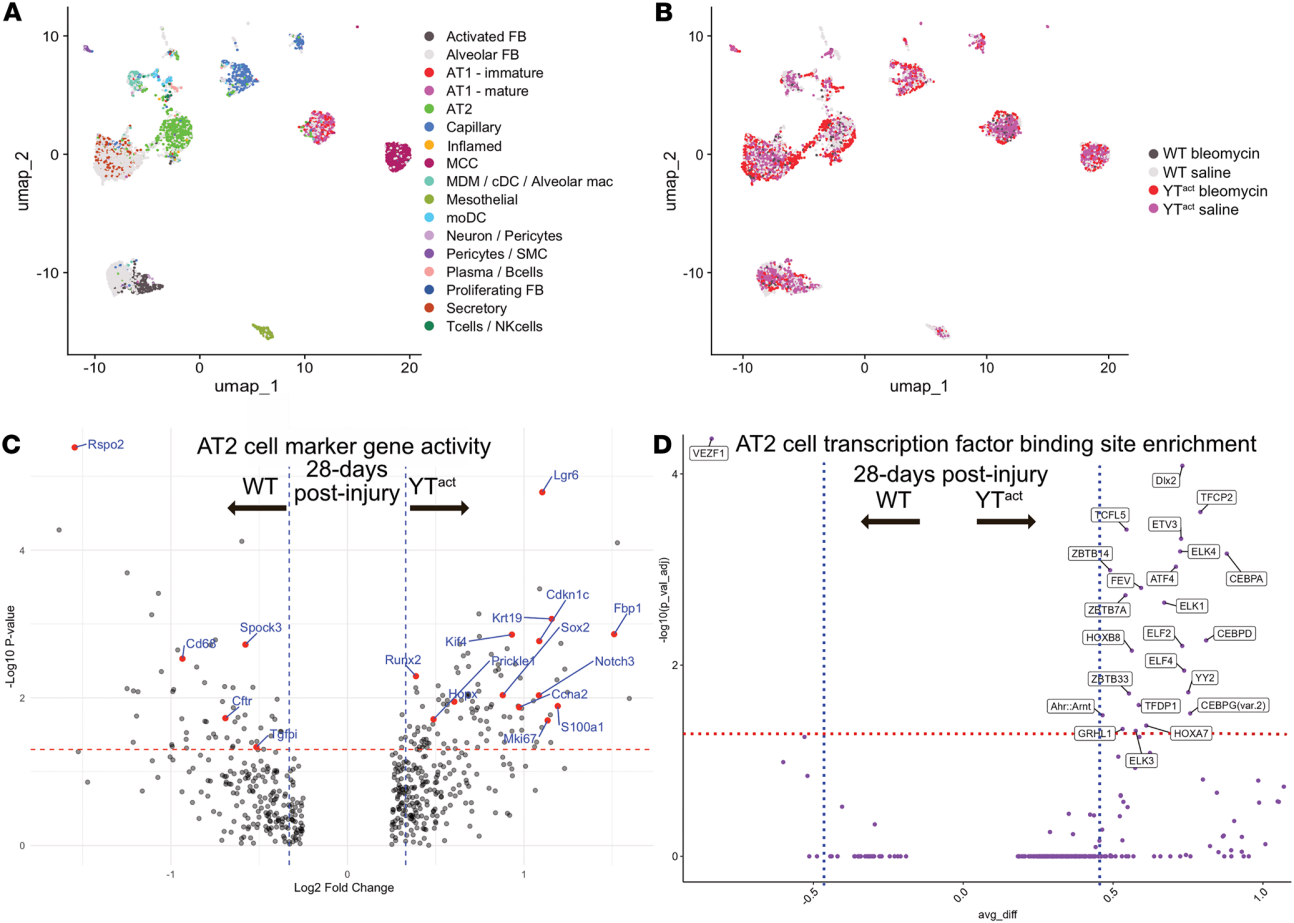
Single-cell ATAC-seq demonstrates altered AT2 cell chromatin accessibility in YT^active^ bleomycin-injured mouse lungs associated with aberrant transitional cells. (**A** and **B**) UMAPs demonstrating clustering from 17 cell types in mouse lungs (**A**) and identifying cells recovered from respective genotype and treatment groups from single-nucleus multiome (RNA + ATAC sequencing [ATAC-seq]) of lung tissue from 28 days after bleomycin (**B**). (**C**) Volcano plot showing upregulated gene activity from AT2 cells of bleomycin-injured WT (left) and YT^active^ (right) mice. (**D**) Single-cell ATAC-seq shows enriched binding sites for open chromatin regions in bleomycin YT^active^ AT2 cells. The “avg_diff” is the fold change computing the average difference in chromVAR *z* score after differential testing between WT (left) and YT^active^ (right) mice.

To examine the mechanisms through which sustained YAP/TAZ activation in AT2 cells prevents functional alveolar repair and fibrosis resolution, we performed single-nucleus multiomic (RNA + ATAC) sequencing of lung tissue from tamoxifen-treated WT and YT^active^ mice 28 days after bleomycin (or saline control). Following data integration, cells were clustered using single-cell ATAC (scATAC) profiles and annotated using canonical marker genes, and the epithelial cell population subset was isolated (Figure 6, A and B, and Supplemental Figure 7A). Analysis of opened chromatin regions in bleomycin-injured AT2 cells found increased gene activity scores of several abnormal genes, including those associated with airway epithelial cells, i.e., *Scgb1a1*, *Muc5b*, *Sox2*, and the AT1/transitional cell markers *Hopx* and *Krt19*, while the AT2-associated transcription factor *Etv5* was reduced (Supplemental Figure 7, B and C) in AT2 cells from YT^active^ bleomycin-injured lungs compared with cells isolated from WT bleomycin-treated lungs. Marker gene analysis using promoter activity demonstrated that genes associated with airway (*Sox2*, *Runx2*), transitional cells (*Hopx*, *Krt19*), and proliferation (*Mki67*, *Ccna2*) were associated with AT2 cells from bleomycin-injured YT^active^ mice (Figure 6C). Analysis of transcription factor binding site enrichment within opened promoter regions was associated with, among others, the AT2 cell regulator *Cebpa* (Figure 6D).

**Figure 7:**
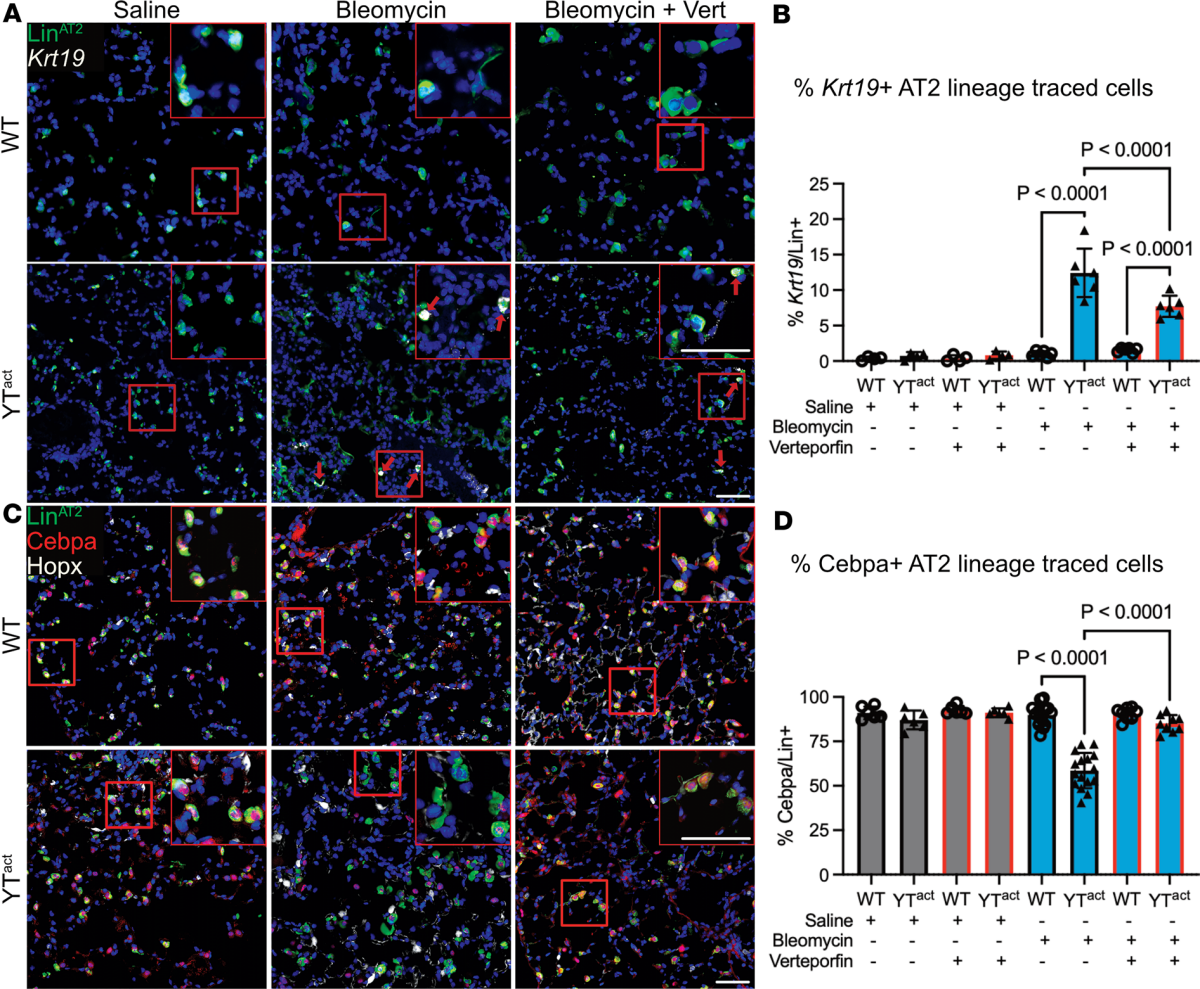
Sustained YAP/TAZ activation leads to aberrant alveolar epithelial cell differentiation and accumulation of transitional cell populations, which is partially corrected by verteporfin treatment. (**A**) Immunofluorescence and RNAscope analysis of *Krt19* (white) and lineage-traced AT2 cells (green). Scale bars: 50 μm. (**B**) Quantification of *Krt19*^+^/lineage-traced cells in each treatment group. Mice across 3 independent experiments were grouped as WT and YT^active^ saline (*n* = 4), WT and YT^active^ saline/verteporfin (*n* = 4), WT and YT^active^ bleomycin (*n* = 6), and WT and YT^active^ bleomycin/verteporfin (*n* = 6). (**C**) Immunofluorescence analysis of AT2 lineage-labeled cells (green), Cebpa^+^ cells (red), and Hopx^+^ AT1 cells (white) in WT and YT^active^ mice given saline, bleomycin, or bleomycin and verteporfin. Scale bars: 50 μm. (**D**) Quantification of Cebpa^+^ nuclei in AT2 lineage-labeled cells. Mice from 3 independent experiments were grouped as WT and YT^active^ saline (*n* = 6), WT and YT^active^ saline/verteporfin (*n* = 6), WT bleomycin (*n* = 14), YT^active^ bleomycin (*n* = 16), WT bleomycin/verteporfin (*n* = 9), and YT^active^ bleomycin/verteporfin (*n* = 9). Ordinary 1-way ANOVA with Šidák’s multiple-comparison test with a single pooled variance was used to determine significance.

To determine whether verteporfin treatment corrected the abnormal AT2 differentiation identified by scATAC-seq, immunofluorescence and RNAscope analysis of lineage-traced AT2 cells was used. RNA-ISH confirmed there were increased lineage-labeled *Krt19^+^* cells (12.4% ± 3.4% *Krt19^+^*Lin^+^) in YT^active^ bleomycin-injured mice compared with WT bleomycin-injured lungs (0.9% ± 0.4% *Krt19^+^*Lin^+^). Verteporfin-treated YT^active^ lungs had significantly reduced *Krt19^+^* cells compared with vehicle-treated YT^active^ bleomycin-injured lungs (7.7% ± 1.5% *Krt19^+^*Lin^+^) (Figure 7, A and B), although this remained higher than in bleomycin-injured verteporfin-treated WT mice (1.6% ± 0.3%). With evidence of altered Cebpa activity from our single-nucleus ATAC-seq results, we then assessed a CEBPA gene module score from human AT2 cells grown in SFFFM (from Figure 2). Genes associated with CEBPA activity were reduced in human epithelial cells cultured with sustained YAP/TAZ activation by XMU-MP-1 (Supplemental Figure 7D). Finally, immunofluorescence analysis of Cebpa in lineage-traced AT2 cells from WT (91.0% ± 3.6%) and YT^active^ (87.1% ± 5.3%) mouse lungs treated with saline or WT bleomycin-treated lungs (89.9% ± 6.0%) demonstrated that Cebpa was readily detected in lineage-traced Sp-C^+^ AT2 cells; however, this was significantly reduced (58.6% ± 9.9%) in AT2 cells in YT^active^ mouse lungs injured with bleomycin. Verteporfin treatment significantly increased Cebpa^+^ lineage-traced cells in the YT^active^ bleomycin-injured lungs (85.1% ± 4.8%) (Figure 7, C and D). Collectively, these findings suggest that sustained YAP/TAZ activity negatively regulates Cebpa or that the sustained YAP/TAZ activation leads to a loss of AT2 cell identity, thereby reducing Cebpa expression. Treatment with verteporfin improved alveolar repair, including decreasing *Krt19^+^* transitional cells as well as restoring Cebpa expression, in lineage-traced AT2 cells. These findings indicate that disruption of aberrant persistent YAP/TAZ activity can promote adaptive repair and potentially delay or stop the progression of pulmonary fibrosis.

**Figure 8:**
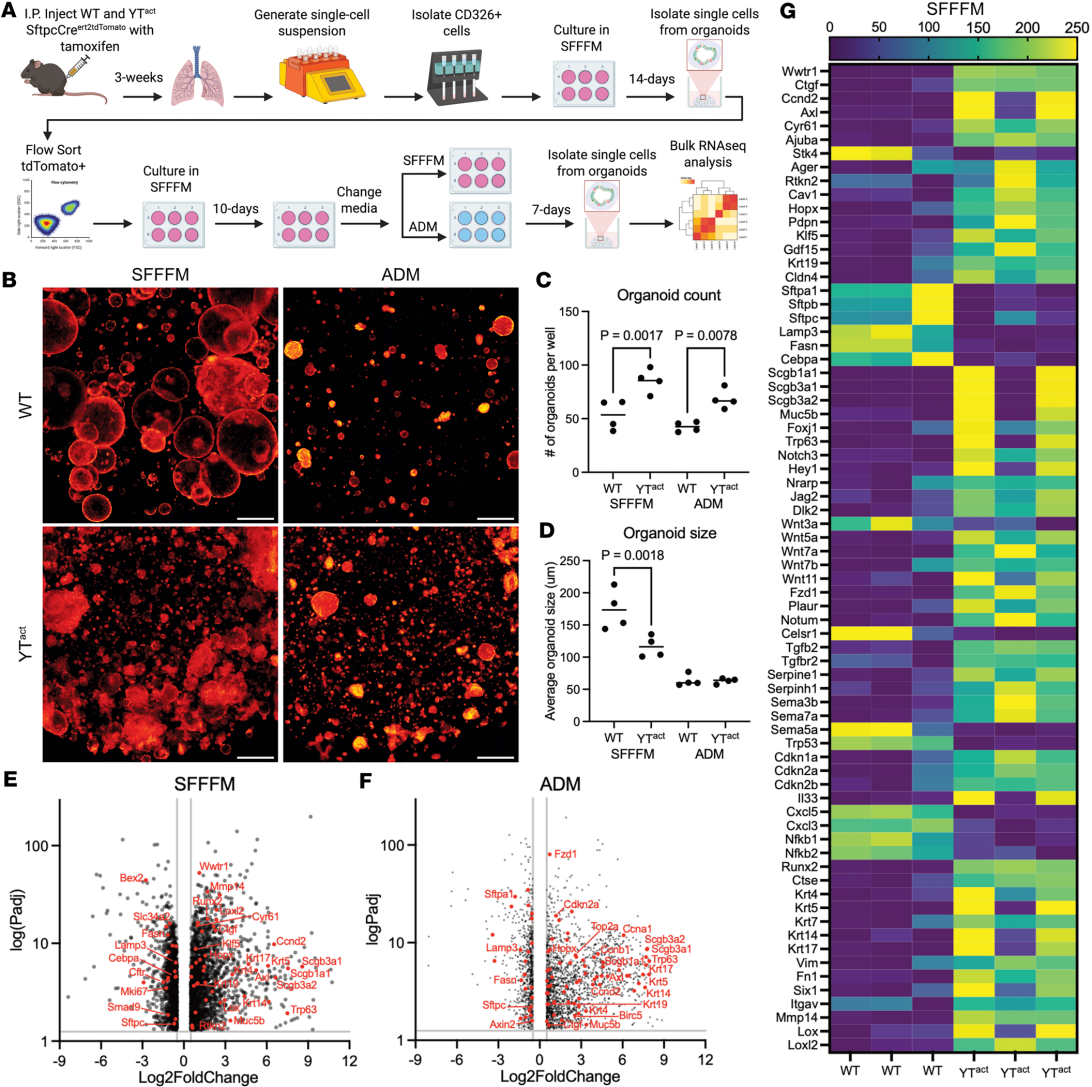
Lineage-traced YT^active^ AT2 cells express markers of aberrant differentiation in feeder-free organoid culture. (**A**) Schematic of experimental design. (**B**) Fluorescent imaging of representative Tomato^+^ lineage-traced organoids at time of collection. Scale bars: 250 μm. (**C**) Quantification of total organoid numbers per well per mouse (*N* = 2 wells per mouse, *N* = 4 mice per group). (**D**) Quantification of average organoid size in each culture condition. Each dot represents an average per mouse. Sequencing and data analysis from 1 of 2 experiments are represented. Statistics determined by ordinary 1-way ANOVA with Šidák’s multiple-comparison test. (**E** and **F**) Volcano plots showing differentially expressed genes (determined using DESeq2) in SFFFM (*N* = 3 mice each genotype) (**E**) and ADM (*N* = 4 mice each genotype) (**F**) medium conditions. Significant-expression cutoffs of *P*_adj_ < 0.05 and log_2_ fold change < –0.5, > 0.5 were used. Positive fold change values indicate increased expression in YT^active^ AT2 cells. (**G**) Heatmap of gene expression patterns from individual mouse organoids cultured in SFFFM showing gene expression associated with YAP/TAZ activation, epithelial cell type markers, developmental pathway markers, and IPF-associated genes.

Next, to determine whether the aberrant AT2 cell differentiation in sustained YT^active^ mice is via cell-autonomous signaling (if YAP/TAZ activation alone in the AT2 cell is sufficient to drive aberrant differentiation), we used the AT2 cell organoid culture model from Katsura et al. (54). *SftpcCre^ert2tdTomato^* WT mice were used as controls for the YT^active^ mice. Mice were injected with 100 mg/kg tamoxifen dissolved in corn oil 3 weeks before cell isolation. Then Cd326^+^ epithelial cells were isolated and cultured in SFFFM for 2 weeks. TdTomato^+^ cells were isolated by FACS and cultured in SFFFM for 10 days. Then cultures were either continued in SFFFM or switched to ADM for 1 week (Figure 8A). YT^active^ organoids cultured in SFFFM or ADM had increased organoid number, while YT^active^ organoids in SFFFM had decreased organoid size, compared with those isolated from WT mice (Figure 8, B–D, and Supplemental Figure 8). These organoids were then dissociated and assessed by bulk RNA-seq to determine transcriptional regulation. YT^active^ organoids had 1,945 genes with increased and 1,409 genes with decreased expression in SFFFM, while 1,812 genes were increased and 704 genes were decreased in ADM, compared with WT AT2 cells. There were 705 shared genes increased and 243 shared genes decreased in the YT^active^ organoids between the 2 medium conditions. YAP/TAZ–associated genes (*Wwtr1*, *Ctgf*, *Ccnd2*, *Axl*, *Cyr61*, and *Ajuba*) were significantly increased along with AT1 differentiation markers including *Ager*, *Rtkn2*, and *Klf5* (Figure 8, E–G). In both SFFFM and ADM, YT^active^ organoids had increased expression of proximal epithelial cell markers including *Scgb1a1*, *Scgb3a2*, *Krt5*, and *Tp63* as well as increased expression of the transitional cell markers *Krt17* and *Krt19*, suggesting loss of normal alveolar cell fate. YT^active^ organoids also had decreased expression of the alveolar epithelial cell markers *Lamp3*, *Fasn*, *Slc34a2*, and *Sftpc*, further supporting this concept. Especially in SFFFM, YT^active^ organoids had increased expression of several genes associated with altered developmental pathway activity. This includes increased Notch (*Notch3*, *Hey1*, *Jag2*), Wnt (*Wnt5a*, *Wnt7b*, *Plaur*), and TGF-β (*Tgfb2*, *Tgfbr2*, and *Serpine1*) signaling. Uncontrolled regulation of developmental pathway signaling is a hallmark of failed repair and has been identified in the epithelium of patients with pulmonary fibrosis (19, 41, 62). Interestingly, most immune-associated genes were downregulated, except for *Il33*, which was increased. Markers of abnormal repair and pulmonary fibrosis were also increased in the YT^active^ mouse organoids, including increased fibroblast activation markers (*Vim*, *FN1*), increased expression of *Mmp14*, and increased expression of the lysyl oxidases *Lox* and *Loxl2*, which are secreted by AT2 cells and are associated with fibroblast activation in pulmonary fibrosis and induced by YAP (63) (Figure 8G).

Collectively, these findings demonstrate that, while previous studies show that YAP/TAZ activity is required for AT2 to AT1 cell differentiation and is required initially to promote repair, sustained unregulated YAP/TAZ activity promotes the aberrant epithelial cell differentiation associated with failed repair, leading to worsened fibrotic remodeling.

## Discussion

Dysregulated repair of the alveolar epithelium is a hallmark of IPF. In this study, we demonstrate that both nuclear YAP and TAZ are increased in SP-C^+^ cells in the IPF lung. This is consistent with our previous work that showed increased YAP activity and loss of MST1/2 in the IPF epithelium (20, 40, 41), and others have shown increased YAP in activated fibroblast populations (39, 42, 44, 45, 64, 65). Sustained YAP activity has also been identified in other forms of pulmonary fibrosis, including Heřmanský-Pudlák syndrome (66). Using a human alveolar organoid model, we found that pharmacologic activation of YT is sufficient to drive AT2 cells into a spectrum of aberrant “transitional” cell morphologies/phenotypes (but, provocatively, not transcriptionally identifiable AT1 cells). This was further studied in a genetic mouse model of YAP/TAZ activation, which demonstrated increased AT1 cell markers but also increased aberrant proximal cell markers and transitional cell markers along with activated developmental pathway signaling. Further, we found that genetic YT activation in AT2 cells worsens experimental lung fibrosis and prevents repair, which could be moderately but significantly ameliorated through a single treatment with a YT inhibitor (verteporfin) even after initiation of injury. Taken together, these results indicate that persistent YT activation in AT2 cells is maladaptive, preventing functional lung repair, and provide additional support for the concept that YT inhibition may be a promising therapeutic approach for IPF in the distal lung.

There have been several prior studies focusing on the role of YAP/TAZ in lung development and injury repair using conditional YAP/TAZ deletion strategies, while to date there has been limited investigation of the long-term impacts of persistent YAP/TAZ activation in the lung epithelium (22, 35–37, 67, 68). In the developing lung, combined deletion of YAP/TAZ using the Sftpc-CreERT2 and Sftpc-rtTA/tetOcre drivers led to reduced numbers of AT1 and AT2 cells (22, 23). Our current study, in concordance with our previous work, showed that YAP was rarely detected in human AT1 cells and only expressed in AT2 cells in IPF, while TAZ was expressed in IPF AT2 cells and murine AT2 cells during repair and was readily detectable in most AT1 cells both during repair and at homeostasis (35, 37). Several studies have demonstrated that YAP and TAZ are essential for AT2 to AT1 differentiation (21–23, 69) and are required for normal repair. Deletion of YAP/TAZ prior to bleomycin, LPS, or bacterial injury results in worsened repair and fibrosis (35–37). Others have shown that deletion of AT2 cell TAZ alone is sufficient to prevent AT2 to AT1 differentiation and increased fibrotic response (38). In addition, deletion of both YAP and TAZ in Hopx^+^ cells resulted in these cells adopting AT2-like features (67), although it does not appear that AT1 cells contribute to repair (70). These studies lead to a paradigm in which YAP/TAZ is initially required for AT2 cell proliferation, while downregulation of YAP/TAZ supports AT2 cell maturation. Without initial YAP/TAZ activity, there is an absence of AT1 transitional cells, and at least TAZ is necessary to drive AT1 cell maturation and TAZ and/or YAP are required for AT1 cell maintenance. This concept shows a need for a finely regulated cell type–specific balance of YAP/TAZ activity during the repair process. Collectively, these findings demonstrate that activation of YAP and/or TAZ is initially essential to guide adaptive alveolar epithelial repair, yet sustained aberrant AT2 activation promotes aberrant differentiation and fibrotic remodeling.

Here, we find evidence that tight regulation of YAP/TAZ activity is essential for proper alveolar repair. We previously showed that YAP activity in AT2 cells was increased early after bleomycin injury with peak nuclear localization at day 7, while TAZ reached peak AT2 expression at 14 days after injury. Both YAP and TAZ returned to near zero activity by 21 days after injury, indicating that YAP and TAZ are dynamically regulated during repair (35). We acknowledge that this observation adds complexity to the growing body of literature in this area, including recent work that implied that Hippo components inhibit alveolar repair (i.e., YAP/TAZ activation may drive adaptive repair) (71). Our current study demonstrates that sustained activation of YAP/TAZ in mouse AT2 cells did not alter the initial injury response or impact fibrosis at 14 days after injury but rather led to exacerbated fibrosis and the presence of persistent transitional cells by 28 days after bleomycin that failed to resolve fibrosis by 8 weeks after injury. Inhibition of this sustained YAP/TAZ activity with a single dose of verteporfin at 14 days was sufficient to moderately but significantly enhance fibrotic resolution in YAP/TAZ–active mice, although it had no effect on WT mice at this time point, further implicating that this was an epithelial YAP/TAZ–driven process. Verteporfin treatment reduced, but not significantly, the number of proliferating lineage-traced cells and did significantly reduce the number of aberrant Sp-C^+^Hopx^+^ lineage-traced cells and the number of Scgb1a1^+^ cells outside the airway. Verteporfin also significantly reduced the number of *Krt19^+^*Lin^+^ abnormal transitional cells and significantly increased the number of Cebpa^+^ lineage-traced cells. There are several potential explanations for these seemingly opposing findings, including differences in the degree and duration of YAP/TAZ inhibition, as well as potentially distinct roles and timing of inhibition/activation of YAP and TAZ in the repair process (37). However, treatment did not completely resolve the presence of these abnormal populations. Recent work in which wild-type mice injured with bleomycin were treated with 45 mg/kg verteporfin (a dose lower than our 60 mg/kg single-dose strategy) every other day from day 7 to day 14 showed that nuclear YAP and the YAP/TAZ target *Ctgf* were inhibited and lung repair was enhanced at day 14 (63). Previous work demonstrated YAP/TAZ activation in lung fibroblasts associated with fibrotic remodeling (39, 42, 44, 65). As reported in a recent preprint, researchers activated YAP in fibroblasts, inhibited the YAP/TAZ activity with 50 mg/kg verteporfin from day 1 to day 21 of bleomycin injury/repair, and found decreased fibroblast activation and enhanced lung repair (72). This demonstrates that verteporfin inhibits both epithelial and fibroblast YAP/TAZ activation. These and our results are consistent with the concept that there is sustained YAP/TAZ activation in IPF, that sustained activation in human or mouse epithelial cells leads to abnormal epithelial cell differentiation, and that pharmacological inhibition of YAP/TAZ may promote adaptive repair.

Further, our in vitro studies demonstrated that YAP/TAZ activation was sufficient to lead to aberrant differentiation of human alveolar epithelial cells into KRT-high and basal-like populations consistent with those seen in IPF (15, 16, 20). These findings are strongly supported by mouse organoids in which YAP and TAZ were genetically activated, leading to the presence of aberrant proximal epithelial cell marker expression, developmental pathway activation, increased genes associated with IPF, and increased transitional marker *Krt19*. Likewise, YAP/TAZ activation resulted in increased *Krt19^+^* transitional cells in mice following bleomycin injury. While the role of *Krt19* is not yet fully elucidated, these intermediate filaments appear as a convergent marker of epithelial cell states associated with injury and maladaptive/fibrotic remodeling (73). This resulted in increased fibrotic remodeling of the mouse lung consistent with a concept that the presence of abnormal persistent transitional cells leads to fibrotic remodeling and the progression of pulmonary fibrosis (74, 75). These transitional cells, whether they initially occur during adaptive repair or are disease emergent, exist in multiple subtypes identified by various Krt markers (*Krt8^+^*, *KRT17^+^*, *KRT5^–^*, *Krt19*^+^) or by various names (DAPTs, PATs) (46, 76–80). The presence of aberrant transitional cells has been identified in multiple forms of pulmonary fibrosis, including fibrosis associated with Heřmanský-Pudlák syndrome (80), spontaneous fibrosis models associated with surfactant protein C mutations (75), and bleomycin-induced models of fibrosis (81, 82) as well as IPF (15–17, 83). Our work has demonstrated that inhibition of the sustained YAP/TAZ activation via verteporfin resolves the presence of these *Krt19^+^* transitional cells that were identified in the mouse lung, which coincided with decreased fibrotic remodeling. Recent work by others demonstrated that removal of other transitional cells — for example, *Krt8*^+^ cells — also resulted in reduced fibrosis (74). Collectively these studies demonstrate that the sustained presence of transitional cell populations, whether from normally occurring transitional cell populations or from failed signaling pathways leading to aberrant differentiation of disease-emergent populations, is associated with fibrotic progression and appears responsive to dynamic regulation of YAP/TAZ activity.

This work also demonstrates that sustained YAP/TAZ activation resulted in opened chromatin associated with Cebpa binding sites. We and others have demonstrated that YAP/TAZ activation leads to opened/altered chromatin accessibility in the lung (22) and other organs, including the heart (84). Generally, opened chromatin and increased presence of transcription factor binding sites are associated with increased activity. However, our immunofluorescent staining of Cebpa in lineage-traced AT2 cells demonstrated decreased nuclear Cebpa localization, and XMU-MP-1–treated (YAP/TAZ–activated via MST1/2 inhibition) human epithelial cells demonstrated decreased gene module score. In mouse organoids with genetic YAP/TAZ activation, *Cebpa* was significantly decreased in SFFFM cultures. These findings imply that YAP/TAZ activity negatively regulates Cebpa. Cebpa was recently shown to be a regulator of AT2 cell fate and maintenance, as activation of Cebpa maintains AT2 cell transcriptional programs, while inhibition of Cebpa is necessary to allow AT2 cell differentiation into AT1 during both development and repair (85, 86). These studies implicate that there may be an inverse relationship between YAP/TAZ and Cebpa in which there is a balance between Cebpa-associated AT2 regulation and AT1 cell YAP/TAZ–associated signaling that regulates alveolar cell fate. These studies are consistent with our findings that YAP/TAZ activation reduced Cebpa and recent work showing reduced alveolar Cebpa in the IPF lung (87), and we have shown YAP/TAZ to be activated in IPF. Inhibition of YAP/TAZ via verteporfin restored Cebpa nuclear localization in lineage-traced AT2 cells, further supporting the concept that Cebpa and YAP/TAZ signaling networks counter-regulate each other.

One limitation of this study is that it does not completely discern whether the effect of verteporfin is mediated predominantly by blocking of the sustained activation of YAP/TAZ in AT2 cells or of both the sustained AT2 cell activation and the previously shown activation of YAP/TAZ in activated fibroblasts (39, 42, 44, 65). While others have reported different findings with verteporfin treatment, our data indicate at minimum that the effects in the epithelium are important contributors to fibrotic remodeling. This work and other recent studies showing that blocking YAP/TAZ enhances repair (63, 72) or that activated YAP/TAZ promotes fibrotic remodeling (37) are in contrast to the established concept that YAP/TAZ activity is also essential initially for adaptive repair and AT2 to AT1 cell differentiation (21–23, 35, 36, 38, 67, 88). As such, targeting YAP/TAZ to enhance repair would require precise timing and potentially may require specific targeting of YAP versus TAZ and cell type–dependent manners, consistent with recent work from others (49, 89). It is possible that some of the effects observed when human alveolar epithelial cells in a reductionist organoid model are treated with XMU-MP-1 may differ from those seen in vivo, where signaling from other cell types may intersect with this pathway. Finally, we acknowledge there may be differential timing or cell type–specific roles of YAP and TAZ during alveolar differentiation and repair, which will require further study.

Cumulatively, our findings demonstrate that the sustained activation of YAP/TAZ via deletion of the Hippo components Stk3 and Stk4 in mice or by inhibition of the human homologs MST1 and MST2 leads to the aberrant AT2 differentiation and persistence of transitional cells. This sustained YAP/TAZ activation results in increased fibrotic remodeling and failed resolution resulting in persistent fibrosis 8 weeks after bleomycin-induced lung injury. A single dose of verteporfin was sufficient to promote resolution of transitional cell fates indicating restored alveolar repair and enhanced resolution of fibrosis in mouse injury models. These findings are consistent with the concept that in fibrotic models where persistent alveolar YAP/TAZ activity is present, attenuating this pathway promotes resolution of fibrotic remodeling and potentially provides a therapeutic target to promote alveolar repair in pulmonary fibrosis.

## Methods

### Sex as a biological variable.

To assess sex as a biological variable, we used equal numbers of male and female mice for all studies. Secondary analysis found no significant difference between male and female mice, and therefore for all subsequent analysis both male and female mice were combined for analyses.

### Human subjects and samples.

Formalin-fixed, paraffin-embedded sections and fresh lung tissue for cell isolation and organoid culture were obtained from deidentified IPF and declined donor lungs removed at the time of lung transplant surgery from the lung tissue repository at Vanderbilt University Medical Center (IRB 060165, 192004). Demographics are shown in Supplemental Table 1.

### Human scRNA-seq reanalysis.

Previously published scRNA-seq from IPF/ILD and control lungs (Gene Expression Omnibus [GEO] GSE227136) was reanalyzed for differential expression of genes in AT2 cells. AT2 cells (including proliferating AT2) from IPF and control samples were a subset from the published integrated dataset, and subject-level pseudobulk differential expression testing was performed using DESeq2 (90).

### Human organoids.

Samples of human donor lungs were dissociated using dispase II (Roche 04942078001), collagenase I (Sigma-Aldrich C0130), and DNase (MilliporeSigma 260913-10MU) in phenol-free DMEM (Gibco 31053028). Cell suspensions were generated in C tubes (Miltenyi Biotec 130-093-237) using a gentleMACS dissociator, then passed through 100 μm and 70 μm filters (MTC Bio C4100 and C4070) to achieve single-cell suspensions. Epithelial cells were isolated by incubation of cells with CD326^+^ microbeads (Miltenyi Biotec 130-061-101) and passing through LS columns (Miltenyi Biotec 130-042-401) positioned on a magnetic stand. CD326^+^ cells were plated in Matrigel droplets (Corning 356231) with serum-free, feeder-free medium (SFFFM) and expanded. Organoids were passaged in bulk, as whole organoids were liberated from Matrigel with 2 mg/mL dispase for 1 hour with gentle disruption and passaged at 1:4 in Matrigel/SFFFM. At initial plating and after each passage, Rock inhibitor was added to the medium for days 1–3. Organoids were then treated with 1 or 3 μm XMU-MP-1 (Tocris 6482) starting at day 4 for YAP/TAZ activation. After 1 week, medium was switched to either SFFFM or alveolar differentiation medium (ADM) for another week, and then organoids were collected for endpoint analysis. For 1 μM and 3 μM comparison, samples were collected 4 days after medium switch.

### Human organoid cell morphology.

Organoids generated from declined donor lungs were grown to confluence in domed Matrigel culture in SFFFM (STEMCELL Alveolar Organoid Media 100-0847), then liberated as whole organoids with dispase (2 mg/mL for 1 hour with gentle disruption after 30 minutes). Organoids were then counted and plated in 10 μL of 1:1 Matrigel/SFFFM at about 350 organoids per well into 96-well μ-Plate angiogenesis imaging plates (ibidi 89646). Organoids were then cultured for 7 days in SFFFM with indicated treatments at day 4 (DMSO or 3 μM XMU-MP-1, MedChemExpress catalog HY-100526), and then transitioned to alveolar differentiation medium (STEMCELL 100-0861) or maintained in SFFFM for 7 days. Samples were then washed with PBS and fixed with 0.4% glutaraldehyde for 20 minutes at room temperature. After 2 washes with PBS, 0.2% NaBH_4_ in dH_2_O was applied and samples were incubated at 4°C for 1 hour for background quenching of autofluorescence of the Matrigel. Wells were then washed 3 times in PBS. A staining solution in PBS-X (0.1% Triton X-100) containing Hoechst (15 μg/mL), SYTO14 (7.5 μM), wheat germ agglutinin conjugated to Alexa Fluor 555 (3.5 μg/mL), and Phalloidin-568 (Thermo Fisher Scientific A12380) (132 nM) was prepared fresh and applied to the wells overnight at 4°C with light excluded. Wells were then washed 3 times for 40 minutes with PBS-X and imaged on an ImageXpress.ai (Molecular Devices) as maximum-intensity projections of 5 μm *Z*-stacks across a depth of 40 μm. All images were collected on the same day under the same settings for the entire plate. Images were analyzed using InCarta (Molecular Devices) with a SINAP custom-trained algorithm for organoid segmentation based on Texas red (phalloidin). After organoid level masking (parent), nuclei were then segmented and analyzed. Standard multivariate analytic parameters within InCarta were used for quantification of organoids across all channels. Nuclei were only analyzed for morphology and RNA characteristics (SYTO14). Data were then analyzed in R using a custom pipeline starting at field-level data (13 fields per well) consisting of metadata assignment, robust normalization, and then median absolute deviation calculation and outlier removal in PCA space following *z* score calculation. Any missing data were then imputed with feature median, and columns with zero variance (failed metrics) were removed. Finally, PCA was performed for display and phenotypic distance analysis in PCA space using Hotelling’s 2-tailed *t* test with adjustment for multiple comparisons.

### Human organoid single-cell isolation and processing for scRNA-seq.

To perform single-cell RNA-seq, human organoids were treated in dispase (2 mg/mL) for 1 hour with gentle disruption after 30 minutes to break up the Matrigel and organoids. Organoids were washed twice in PBS and spun down at 500*g*. Organoids were then treated with 1 mL trypsin (0.05%) for 7 minutes, then washed in PBS containing 5% BSA, washed 2 more times in PBS, then passed through 100 μm, then 70 μm cell filters to generate single-cell suspensions. Samples were pooled across donors per treatment group. We then used 10x Genomics scRNA-seq to target 10,000 cells per group, with 50,000 reads per cell. Donors were genetically demultiplexed using Demuxafy (91) and the Vireo pipeline (92). Clustering was performed and identified with marker genes using Seurat/Signac pipeline (93, 94).

### Animal husbandry and YAP/TAZ gene regulation.

These studies were approved by the IACUC at Vanderbilt University Medical Center. *SftpcCre^ert2(blh)^ Stk3^fl/fl^Stk4^fl/fl^* mice (The Jackson Laboratory 028054 [ref. 95] crossed with The Jackson Laboratory 017635 [ref. 96]) were crossed with *Stk3^fl/fl^Stk4^fl/fl^* mice to generate experimental cohorts (YT^active^), with Cre^–^ littermates used as wild-type (WT) controls. *SftpcCre^ert2rosatdTomato^ Stk3^fl/fl^Stk4^fl/fl^* mice crossed with *Stk3^fl/fl^Stk4^fl/fl^* mice served as experimental cohorts for lineage-traced studies, with *SftpcCre^ert2rosatdTomato^* mice (The Jackson Laboratory 028054 [ref. 95] crossed with The Jackson Laboratory 007909 [ref. 97]) used as lineage-traced WT controls. Mice were given tamoxifen (MilliporeSigma T5648) dissolved in corn oil via i.p. injection at 100 mg/kg either 3 weeks before or 2 weeks after bleomycin injury to induce YAP/TAZ activation and lineage tracing. For YAP/TAZ inhibition experiments, verteporfin (Cell Signaling 64260) dissolved in DMSO was administered via i.p. injection at 60 mg/kg 2 weeks after injury.

### Lung injury and collection.

Bleomycin (0.08 IU) was suspended in sterile saline and administered intratracheally at 100 μL volume with equal volumes of saline administered as controls. Mice were sacrificed at indicated time points. The right lung was inflation-fixed with 10% buffered formalin at 25 cm H_2_O pressure, excised, and submerged in 10% buffered formalin. The left lung was flash-frozen in liquid nitrogen and processed for Sircol collagen analysis or for nucleus isolation for analysis.

### IHC analysis.

Inflation-fixed mouse lungs (as described above) and organoids were fixed overnight at 4°C, then processed and embedded in paraffin. Human lung samples were collected from deidentified transplant lungs from IPF patients, and collected donor lungs were rejected for transplant. All histological staining was done on 5 μm sections, and immunofluorescence and/or RNAscope RNA-ISH was conducted with antibodies and RNA probes listed in Supplemental Table 2. Citrate Buffer (pH 6; Sigma-Aldrich C9999) was used for antigen retrieval, and then slides were washed with deionized water. Slides were covered in block buffer (5% BSA) for 1 hour at room temperature. Block was removed, and primary antibodies were added and allowed to incubate overnight at 4°C. Slides were washed 3 times in PBST, and secondary antibody was added and allowed to incubate for 2 hours at room temperature. For human lung samples, Vector TrueVIEW (Vector Laboratories catalog SP-8400) was added per the manufacturer’s instructions for 5 minutes to quench autofluorescence of red blood cells and collagen. Slides were washed again 3 times in PBST, and coverslips were applied with mounting medium. Slides were imaged capturing 10 non-overlapping frames per lung with a ×20 objective on a Keyence BZ-X710 inverted fluorescence microscope.

### Fibrosis scoring.

Semiquantitative analysis of fibrosis was done using modified Ashcroft scoring (98) of Masson’s trichrome–stained slides. For each lung, 2 independent blinded scorers assessed ten ×20 objective magnification frames per lung, and scores were averaged. For scoring discrepancy of greater than 1.5, scores were discussed, and consensus was reached. Collagen content was quantified using a Sircol soluble and insoluble collagen assay kit (Biocolor S5000).

### Single-cell multiome sequencing and analysis.

Single-nucleus multiome (assay for transposase-accessible chromatin [ATAC] + RNA) sequencing was performed using 10x Genomics Chromium sequencing targeting 10,000 nuclei per sample and 50,000 reads per nuclei. Single nuclei were isolated from flash-frozen left lung tissue pooling one male and one female mouse per group, resulting in a total of 21,144 nuclei identified via alignment to the GRcm39 reference genome. Gene expression mapped to the genome with relatively low confidence/read depth. SoupX (99) and scDoublet (100) were used to remove ambient DNA and detect doublets. Multi-resolution clustering, normalization, and dimension reduction were performed with Seurat/Signac (93, 94). The identity of 17 cell types was determined with marker genes from other in-house mouse lung scRNA-seq maps. The scATAC-seq data were mapped to these clusters by identification of open chromatin regions associated with specific marker genes and integrated with the scRNA-Seq with Harmony (101). Plots were made using ggplot2 (102) and scCustomize (103). For scATAC-seq analysis, MACS2 (104) was used for peak calling. Gene activity scores were generated using peaks within 2 kb upstream of the transcriptional start sites. Promoter-associated transcription factor enrichment was quantified using chromVAR motif analysis in Signac (105).

### Mouse AT2 cell organoid cultures and RNA-seq analysis.

To induce recombination/lineage labeling, *SftpcCre^ert2rosatdTomato^* WT and *SftpcCre^ert2rosatdTomato^ Stk3^fl/fl^Stk4^fl/fl^* YT^active^ mice were injected with 3 mg/mouse tamoxifen dissolved in corn oil. After 3 weeks to allow tamoxifen washout, mice were sacrificed (*N* = 4 per genotype), lungs were excised, single-cell suspensions were generated similarly to the human lung protocol above, and Cd326^+^ epithelial cells were isolated using Miltenyi Biotec magnetic bead separation. Cells were cultured following the Katsura et al. 2020 (54) protocol in Matrigel droplets (100 mL droplet 50:50 SFFFM/Matrigel with 50,000 cells per droplet) in mouse serum-free, feeder-free medium (SFFFM) for 14 days. Then cells were liberated from Matrigel droplets using 2 mg/mL dispase (45 minutes), followed by single-cell suspensions via trypsin for 7 minutes. Cells were washed and passed through a 40 μM filter, then incubated with live/dead stain (calcein violet live stain, eBioscience) (30 minutes, then washed 3 times), followed by flow sorting of live tdTomato^+^ AT2 cells. Sorted AT2 cells were then replated in Matrigel droplets as above (10,000 cells per droplet) and cultured for 10 days in SFFFM, after which organoids were either continued in SFFFM or switched to alveolar differentiation medium (ADM) and cultured an additional 7 days. Organoids were then imaged (for quantification of organoid size and number), released from Matrigel, and processed for bulk RNA-seq transcriptional analysis. In short, cells were lysed and RNA was extracted using an RNeasy Plus Mini Kit (QIAGEN catalog 74134). ERCC spike-in (ThermoFisher) was added to normalized RNA (500 ng/sample), and then the NEBNext Ultra II RNA Library Prep Kit with poly(A) mRNA magnetic isolation module was used to generate cDNA and sequenced (GENEWIZ) on a NovaSeq X platform targeting 30 million paired-end reads (2 × 150 bp) per sample. After sequencing, adapters were trimmed with Trimmomatic, reads were mapped to the mouse GRCm39 genome with STAR aligner (106) (v2.5.2b), and gene hit counts within exon regions were determined using the Counts function. Differential expression and analysis were performed with DESeq2 (90). Genes with absolute fold change greater than 0.5 and adjusted *P* value less than 0.05 were considered differentially expressed. Volcano plots were generated with positive fold changes indicating increased expression in YT^active^ AT2 cells compared with WT AT2 cells cultured in the indicated media.

### Statistics.

Statistical analysis was performed using GraphPad Prism 10. Normality was tested using Shapiro-Wilk and Kolmogorov-Smirnov tests. Unless otherwise noted, for 2-variable comparisons, 2-tailed unpaired parametric *t* tests were used. For comparisons with more than 2 variables, ordinary 1-way ANOVA with Šidák’s multiple-comparison test with a single pooled variance was used for normal distribution. For all bar graphs with individual points labeled, dots represent individual mice as an average of multiple fields or organoid droplets unless otherwise noted.

### Study approval.

Mouse studies were approved by Vanderbilt University Medical Center’s IACUC, under approval M1500027 and M2400059. Deidentified human lung samples were acquired, and human studies were approved under protocols 060165 and 192004 by Vanderbilt University Medical Center’s human subjects IRB.

### Data availability.

Genomic data are available for download at GEO GSE326359 (human data) and GSE327686 (mouse data), and analysis script is available at Github (https://github.com/KropskiLab/Gokey-2025_yaptaz_activation; commit ID 8e16af4e8ddca2e281ec30ad84d969ba797fb5a2).

## Author contributions

IPG, ASM, JAK, and JJG designed research studies. IPG, ASM, NMG, ACC, GTD, TPS, UKS, HED, APS, and JJG conducted experiments. IPG, ASM, NMG, GTD, TPS, DSN, and JJG acquired data. IPG, ASM, ACC, JPC, SS, SSG, TSB, JAK, and JJG analyzed data. IPG, ASM, JAK, and JJG wrote the manuscript. IPG and ASM share first author of this manuscript. Both contributed heavily to this study. IPG initiated the project and performed mouse studies/analysis, and as the project initiator, is listed first. ASM performed human cell culture studies and associated analysis including scRNA and cell painting All authors edited the manuscript.

## Conflict of interest

JAK reports grants/contracts from Boehringer Ingelheim and Bristol Myers Squibb and consulting for Boehringer Ingelheim.

## Funding support

This work is the result of NIH funding, in whole or in part, and is subject to the NIH Public Access Policy. Through acceptance of this federal funding, the NIH has been given a right to make the work publicly available in PubMed Central.

- NIH/National Heart, Lung, and Blood Institute (NHLBI), R01HL176912 to JJG.
- NIH/NHLBI R01HL145272 and R01HL153246 to JAK and R01HL151016 to TSB.
- Vanderbilt Faculty Research Scholars to JJG and ASM.
- Francis Family Foundation to JJG and ASM.
- Pulmonary Fibrosis Foundation Fellowship to ASM.
- Vanderbilt Institute for Clinical Translational Research VR55627 and VR55246 under Clinical and Translational Science Award 5UL1TR002243 to JJG.

## Supplementary Material

Supplemental data

Supplemental table 2

Supporting data values

## Version 1 06/09/2026

In-Press Preview

## Version 2 08/10/2026

Electronic publication
