# Supplementary material for: Sustained YAP/TAZ activation promotes aberrant alveolar epithelial cell differentiation and drives persistent fibrotic remodeling
Source: JCI Insight. 2026 Jun 9;11(15):e198113. doi: 10.1172/jci.insight.198113 (PMC13464018; doi:10.1172/jci.insight.198113)
Supplement: Supplementary file 1 [file jciinsight-11-198113-s283.pdf]

Supplemental Table 1: Patient data including age and sex of donor lungs rejected for transplant and IPF lungs used for analysis of YAP/TAZ expression and organoids associated with Figures 1-3.

Supplemental Table 2: Antibodies and RNAscope probes used, along with catalog number, supplier, and concentrations used.

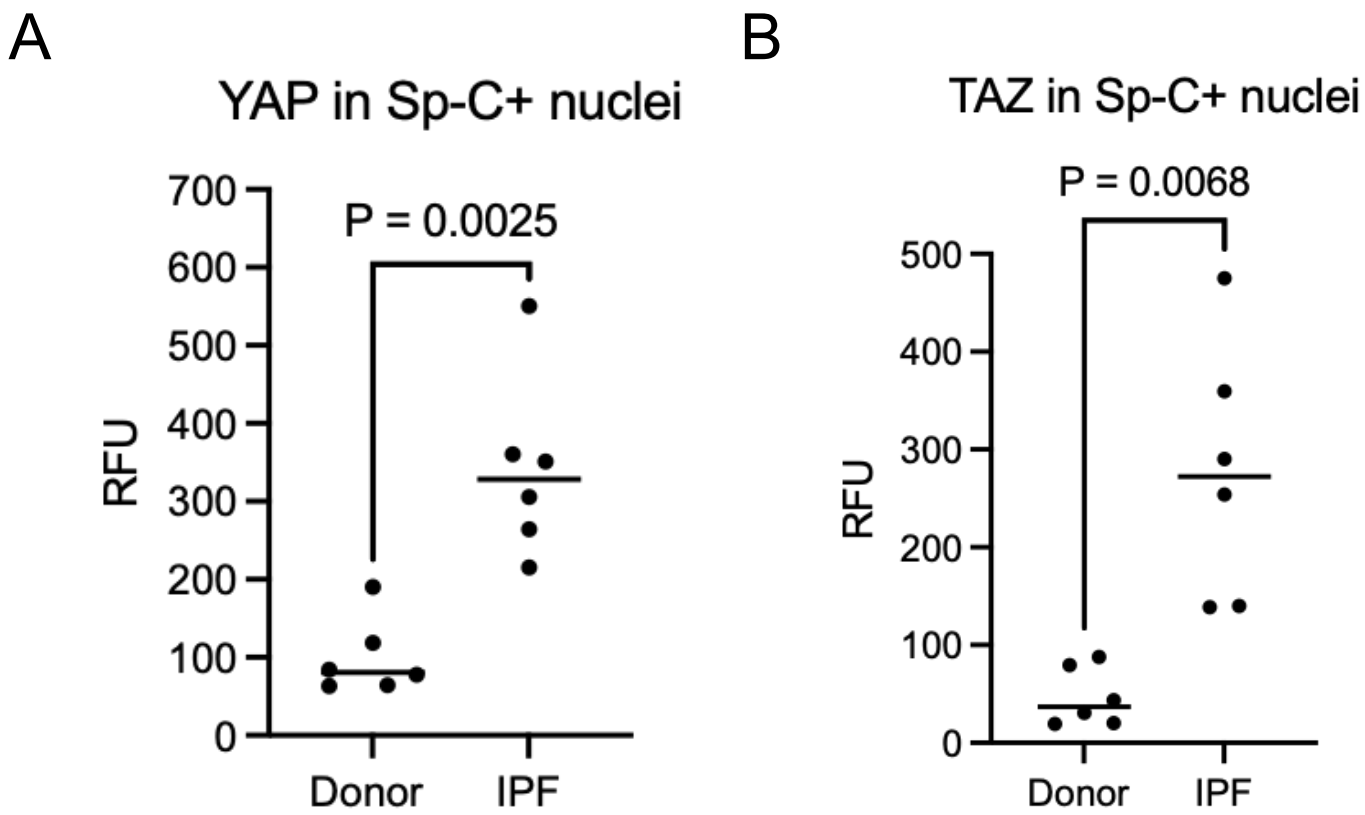
A
YAP in Sp-C+ nuclei
P = 0.0025
RFU
700
600
500
400
300
200
100
0
Donor
IPF
B
TAZ in Sp-C+ nuclei
P = 0.0068
RFU
500
400
300
200
100
0
Donor
IPF

Supplemental Figure S1: Quantification of relative fluorescence intensity of YAP (**A**) and TAZ (**B**) in Sp-C+ DAPI+ nuclei in IPF and healthy donors. Statistics determined using Welch's t test. Supplemental figure associated with main Figure 1.

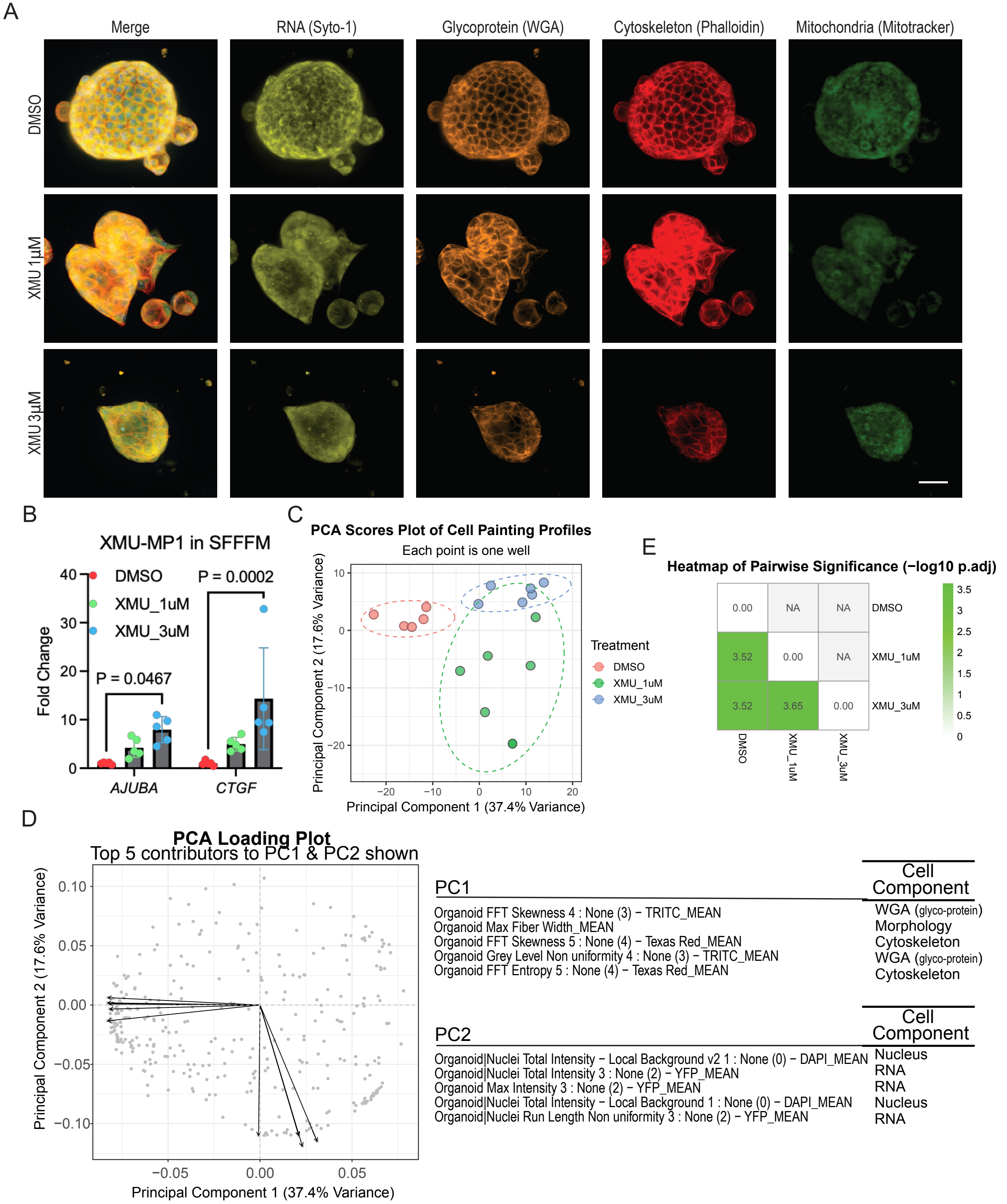
A
Merge
RNA (Syto-1)
Glycoprotein (WGA)
Cytoskeleton (Phalloidin)
Mitochondria (Mitotracker)
DMSO
XMU 1µM
XMU 3µM
B
XMU-MP1 in SFFFM
DMSO
XMU_1uM
XMU_3uM
P = 0.0002
P = 0.0467
Fold Change
AJUBA
CTGF
C
PCA Scores Plot of Cell Painting Profiles
Each point is one well
Principal Component 2 (17.6% Variance)
Principal Component 1 (37.4% Variance)
Treatment
DMSO
XMU_1uM
XMU_3uM
E
Heatmap of Pairwise Significance (−log10 p.adj)
0.00 NA NA DMSO
3.52 0.00 NA XMU_1uM
3.52 3.65 0.00 XMU_3uM
DMSO
XMU_1uM
XMU_3uM
D
PCA Loading Plot
Top 5 contributors to PC1 & PC2 shown
Principal Component 2 (17.6% Variance)
Principal Component 1 (37.4% Variance)
PC1
Cell Component
Organoid FFT Skewness 4 : None (3) − TRITC_MEAN
Organoid Max Fiber Width_MEAN
Organoid FFT Skewness 5 : None (4) − Texas Red_MEAN
Organoid Grey Level Non uniformity 4 : None (3) − TRITC_MEAN
Organoid FFT Entropy 5 : None (4) − Texas Red_MEAN
WGA (glyco-protein)
Morphology
Cytoskeleton
WGA (glyco-protein)
Cytoskeleton
PC2
Cell Component
Organoid|Nuclei Total Intensity − Local Background v2 1 : None (0) − DAPI_MEAN
Organoid|Nuclei Total Intensity 3 : None (2) − YFP_MEAN
Organoid Max Intensity 3 : None (2) − YFP_MEAN
Organoid|Nuclei Total Intensity − Local Background 1 : None (0) − DAPI_MEAN
Organoid|Nuclei Run Length Non uniformity 3 : None (2) − YFP_MEAN
Nucleus
RNA
RNA
Nucleus
RNA

Supplemental Figure S2: A) Representative images of Cell painting single channel images of organoids with an escalating dose of XMU-MP-1. Scale bars are 50μm. **B)** Gene expression analysis (quantitative qPCR) of *AJUBA* and *CTGF* in XMU-MP1 treated organoids cultured in SFFFM demonstrating increased YAP/TAZ activity in epithelial cells isolated from 5 donor lungs. Statistics determined using 2-way ANOVA with Sidak's multiple comparison test. **C.)** PCA plot of cell painting profiles from Vehicle (DMSO), 1μM XMU-MP-1, or 3 μM XMU-MP-1. **D.)** PCA loadings plot highlighting the top five contributors for PC 1 and 2 as well as their respective general category of cell component based on cell compartment stained. **E.)** Statistical analysis of phenotypic distance between groups. P-values obtained from Hotelling's T-test in PCA space from all replicates. Supplemental figure associated with main Figure 2.

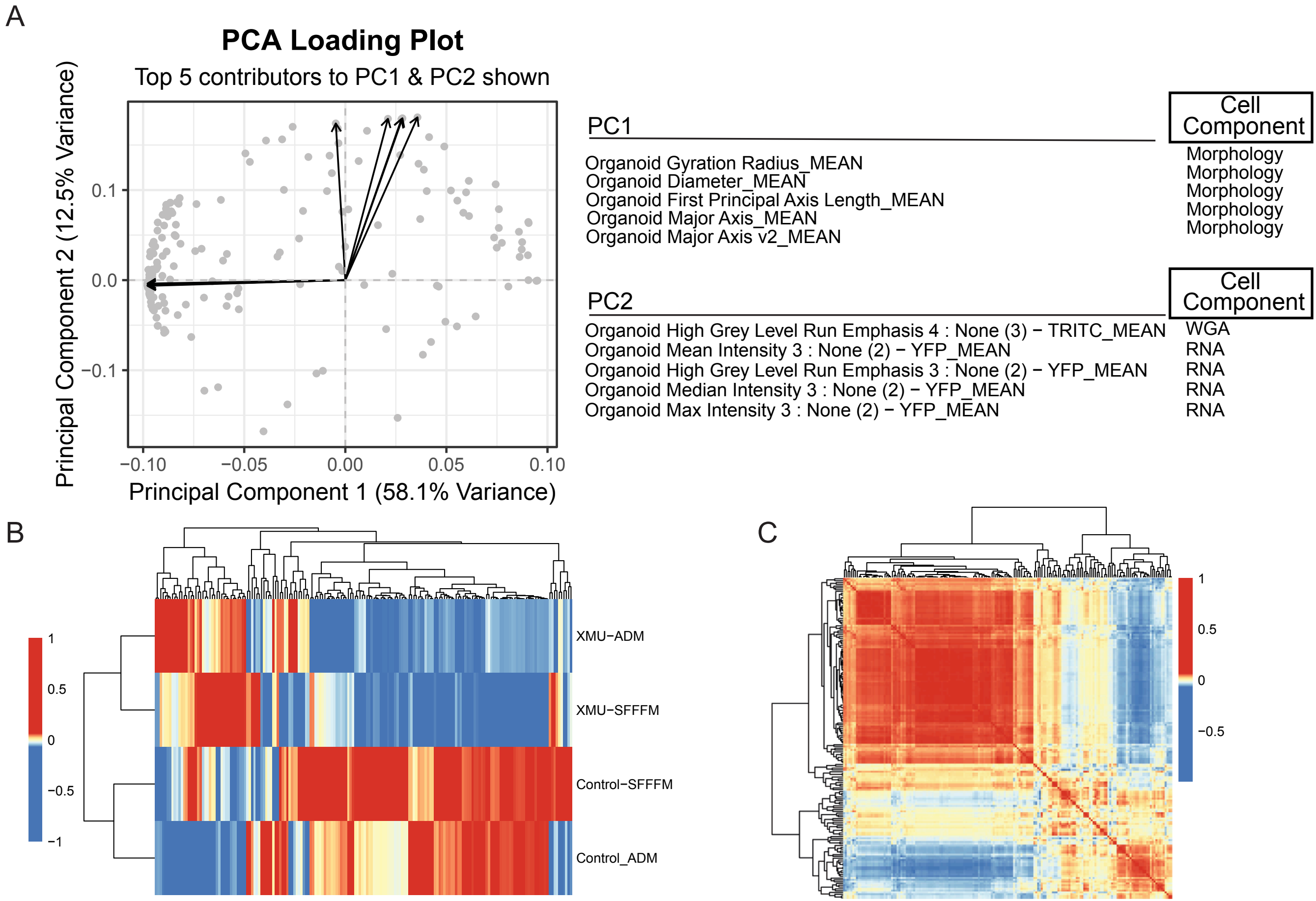
A
PCA Loading Plot
Top 5 contributors to PC1 & PC2 shown
Principal Component 2 (12.5% Variance)
Principal Component 1 (58.1% Variance)
0.1
0.0
−0.1
−0.10
−0.05
0.00
0.05
0.10
PC1
Cell Component
Organoid Gyration Radius_MEAN Morphology
Organoid Diameter_MEAN Morphology
Organoid First Principal Axis Length_MEAN Morphology
Organoid Major Axis_MEAN Morphology
Organoid Major Axis v2_MEAN Morphology
PC2
Cell Component
Organoid High Grey Level Run Emphasis 4 : None (3) − TRITC_MEAN WGA
Organoid Mean Intensity 3 : None (2) − YFP_MEAN RNA
Organoid High Grey Level Run Emphasis 3 : None (2) − YFP_MEAN RNA
Organoid Median Intensity 3 : None (2) − YFP_MEAN RNA
Organoid Max Intensity 3 : None (2) − YFP_MEAN RNA
B
1
0.5
0
−0.5
−1
XMU−ADM
XMU−SFFFM
Control−SFFFM
Control_ADM
C
1
0.5
0
−0.5

Supplemental Figure S3: A) PCA loadings plot highlighting the top five contributors for PCA 1 and 2 as well as their respective general category of cell component based on cell compartment stained from PCA analysis presented in main Figure 2. **B**) Hierarchically clustered and scaled heatmap of all extracted features by media condition **C**) Correlation diagram of extracted features, scaled by Pearson correlation value to demonstrate underlying data structure.

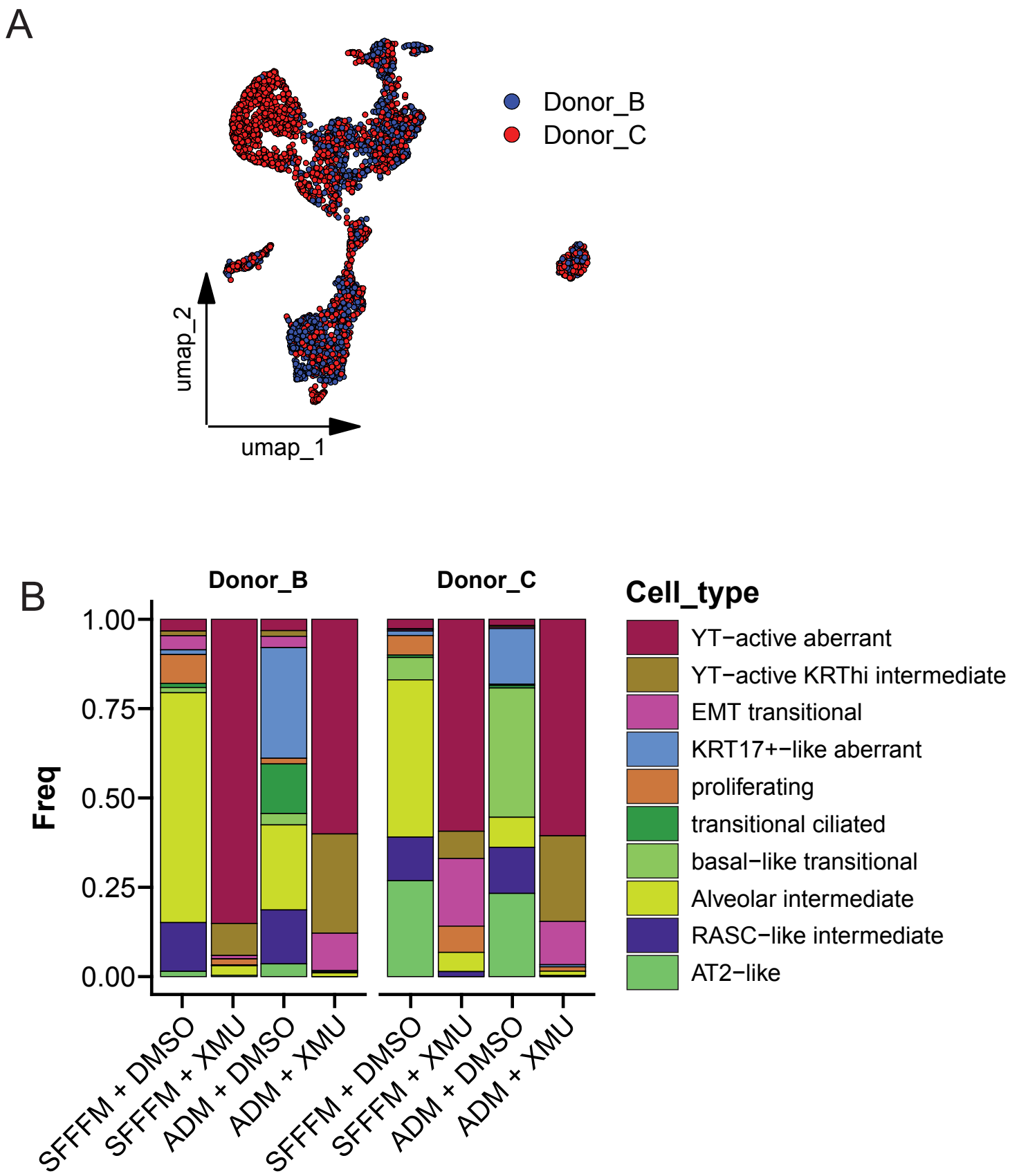

A
Donor_B
Donor_C
umap_2
umap_1
B
Donor_B
Donor_C
Freq
1.00
0.75
0.50
0.25
0.00
SFFFM + DMSO
SFFFM + XMU
ADM + DMSO
ADM + XMU
Cell_type
YT−active aberrant
YT−active KRThi intermediate
EMT transitional
KRT17+−like aberrant
proliferating
transitional ciliated
basal−like transitional
Alveolar intermediate
RASC−like intermediate
AT2−like

Supplemental Figure S4: A) UMAP embedding of human organoid scRNA data in main Figure 3 separated by Donor **B**) Stacked bar chart of annotated cell types separated by Donor and media by drug permutations demonstrating conserved response to XMU agonism across biologic donors.

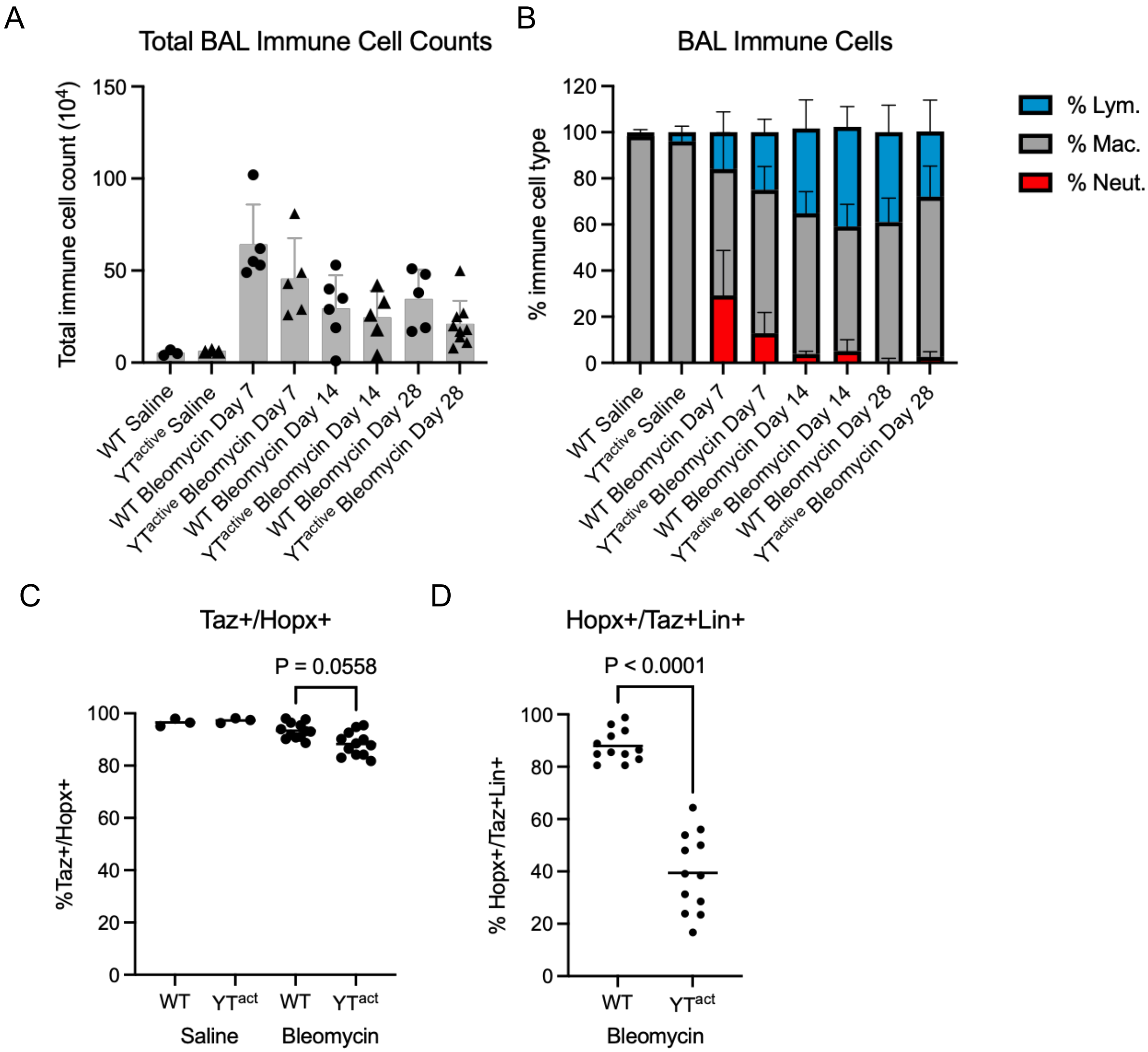

A
Total BAL Immune Cell Counts
Total immune cell count (10^4)
150
100
50
0
WT Saline
YT^active Saline
WT Bleomycin Day 7
YT^active Bleomycin Day 7
WT Bleomycin Day 14
YT^active Bleomycin Day 14
WT Bleomycin Day 28
YT^active Bleomycin Day 28
B
BAL Immune Cells
% immune cell type
120
100
80
60
40
20
0
WT Saline
YT^active Saline
WT Bleomycin Day 7
YT^active Bleomycin Day 7
WT Bleomycin Day 14
YT^active Bleomycin Day 14
WT Bleomycin Day 28
YT^active Bleomycin Day 28
% Lym.
% Mac.
% Neut.
C
Taz+/Hopx+
P = 0.0558
%Taz+/Hopx+
100
80
60
40
20
0
WT
YT^act
WT
YT^act
Saline
Bleomycin
D
Hopx+/Taz+Lin+
P < 0.0001
% Hopx+/Taz+Lin+
100
80
60
40
20
0
WT
YT^act
Bleomycin

Supplemental Figure S5: Total quantification (**A**) and DiffQuick differential immune cell type quantification (**B**) of broncho-alveolar lavage fluid from WT and YT^{active} mice subjected to either saline or bleomycin injury collected at 7, 14, and 28-days post bleomycin injury. **C)** Quantification of the percentage of Hopx+ cells that expressed nuclear Taz+. **D)** Quantification of Taz+ lineage traced cells expressing nuclear Hopx. Statistics determined using non-parametric Two-tailed Mann-Whitney test. Supplemental figure is associated with main Figure 4.

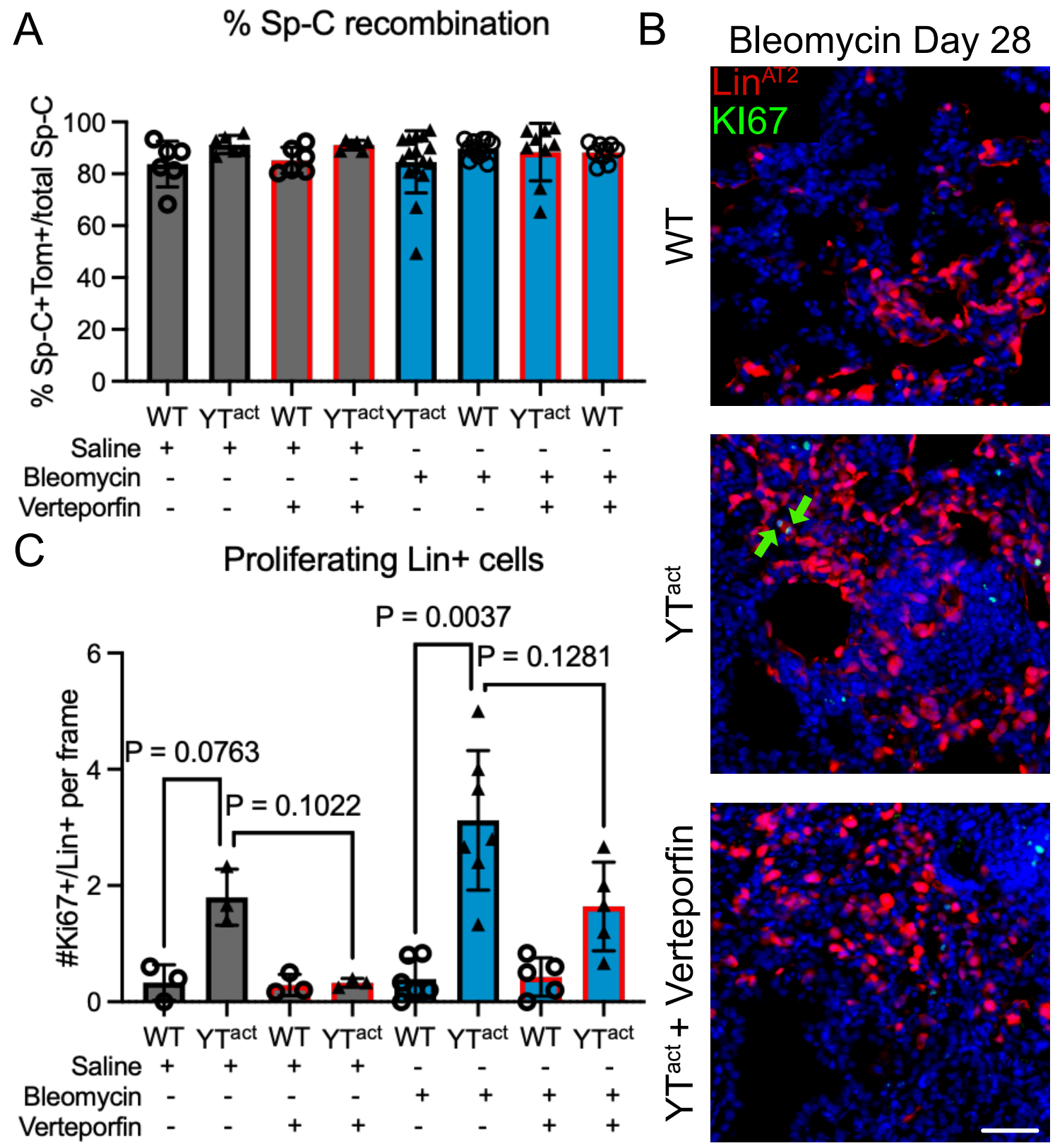

A
% Sp-C recombination
% Sp-C+Tom+/total Sp-C
100
80
60
40
20
0
WT YTact WT YTact YTact WT YTact WT
Saline + + + + - - - -
Bleomycin - - - - + + + +
Verteporfin - - + + - - + +
B
Bleomycin Day 28
LinAT2
KI67
WT
YTact
YTact + Verteporfin
C
Proliferating Lin+ cells
#Ki67+/Lin+ per frame
6
4
2
0
P = 0.0763
P = 0.1022
P = 0.0037
P = 0.1281
WT YTact WT YTact WT YTact WT YTact
Saline + + + + - - - -
Bleomycin - - - - + + + +
Verteporfin - - + + - - + +

Supplemental Figure S6: A) Quantification of Sp-C$^+$/Lineage traced cells to determine recombination efficiency of the mouse model. **B)** Immunofluorescence analysis of Ki67+ (green) proliferating lineage traced AT2 cells (red) at 28-days post-bleomycin injury. Green arrows point to proliferating lineage traced cells. **C)** Quantification of the percentage of proliferating cells per frame. Each dot indicates an average per mouse. Scale bar is 50μm. Supplemental figure is associated with main Figure 5.

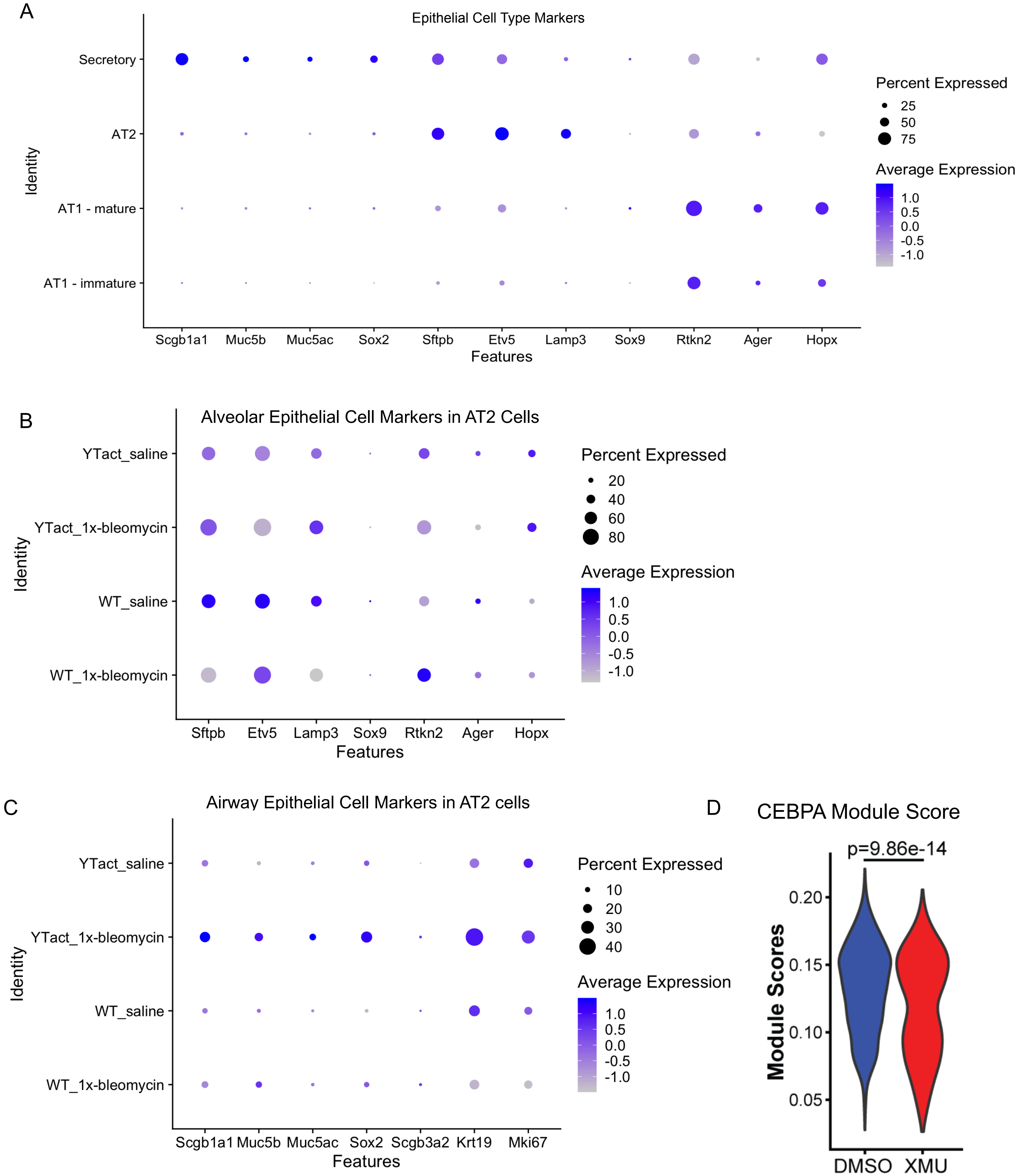

A
Epithelial Cell Type Markers
Secretory
AT2
AT1 - mature
AT1 - immature
Identity
Scgb1a1
Muc5b
Muc5ac
Sox2
Sftpb
Etv5
Lamp3
Sox9
Rtkn2
Ager
Hopx
Features
Percent Expressed
25
50
75
Average Expression
1.0
0.5
0.0
-0.5
-1.0
B
Alveolar Epithelial Cell Markers in AT2 Cells
YTact_saline
YTact_1x-bleomycin
WT_saline
WT_1x-bleomycin
Identity
Sftpb
Etv5
Lamp3
Sox9
Rtkn2
Ager
Hopx
Features
Percent Expressed
20
40
60
80
Average Expression
1.0
0.5
0.0
-0.5
-1.0
C
Airway Epithelial Cell Markers in AT2 cells
YTact_saline
YTact_1x-bleomycin
WT_saline
WT_1x-bleomycin
Identity
Scgb1a1
Muc5b
Muc5ac
Sox2
Scgb3a2
Krt19
Mki67
Features
Percent Expressed
10
20
30
40
Average Expression
1.0
0.5
0.0
-0.5
-1.0
D
CEBPA Module Score
p=9.86e-14
Module Scores
0.20
0.15
0.10
0.05
DMSO
XMU

Supplemental Figure S7: A) Dotplot of cell type markers of the 4 epithelial cell types identified by our single nucleus multiome (RNA^{+} ATACseq) analysis associated with Figure 5. **B**) Dotplot showing AT2 cell associated gene activity scores and **C**) Dotplot showing airway, transitional, and proliferation marker gene activity scores determined from single-cell ATAC sequencing associated with Figure 6. **D**) Gene module score showing decreased signaling associated with CEBPA activity from scRNAseq of human organoids from Figure 3.

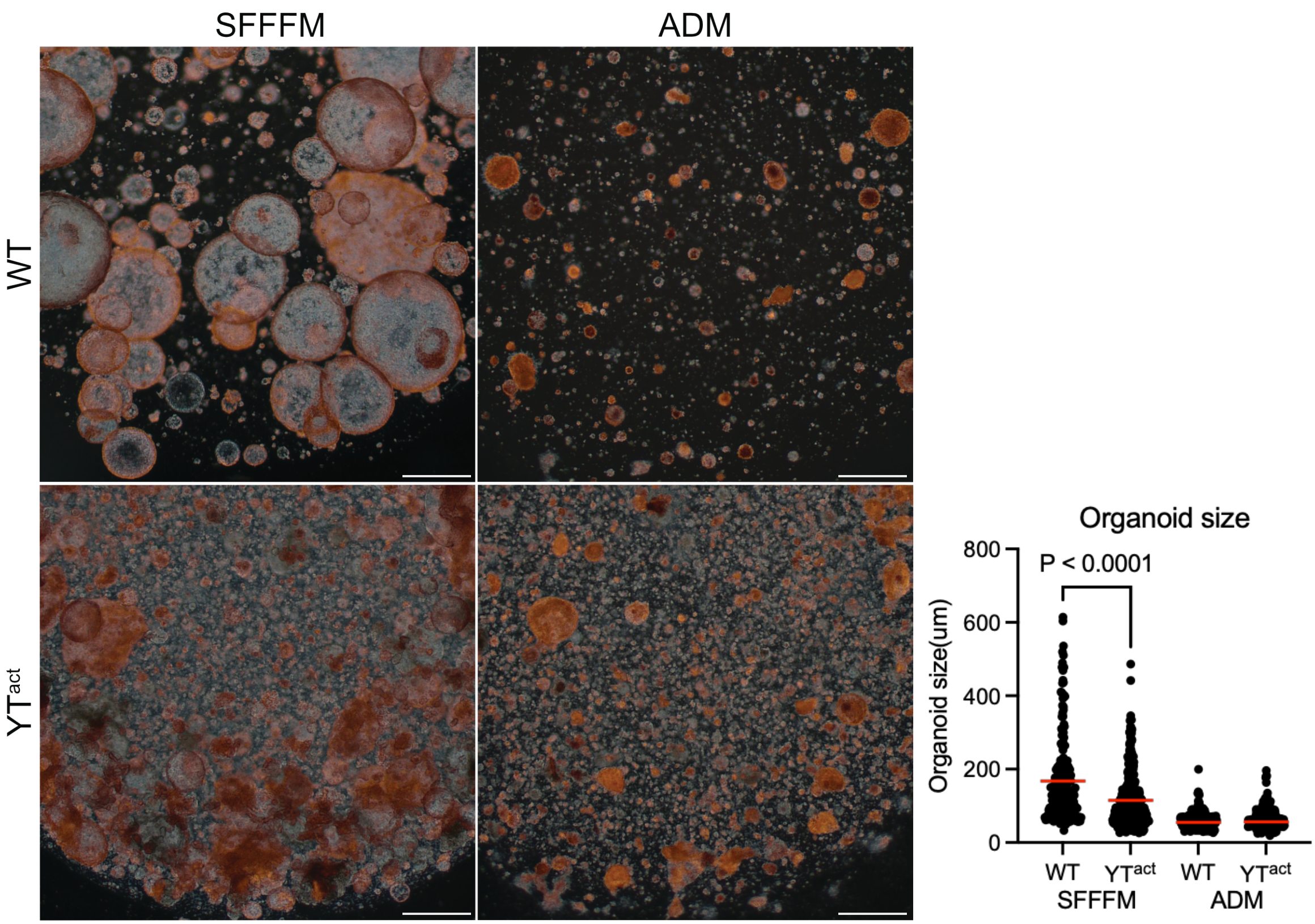
SFFFM
ADM
WT
YTact
Organoid size
P < 0.0001
Organoid size(um)
800
600
400
200
0
WT
YTact
WT
YTact
SFFFM
ADM

Supplemental Figure S8: Overlayed light and fluorescent (TdTomato) imaging of mouse AT2 organoids cultured in SFFFM and ADM. Quantification of organoid size represented as individual organoids. Red line represents mean value. Organoid analysis was limited to organoids >25μm in size. Supplemental figure is associated with main Figure 8.

Sample	Lung state	Age	Smoking Status	COD	Notes
VUHD84	Healthy Donor	34/Female	1p/d/7 years	CPA	
VUHD85	Healthy Donor	53/Male	unknown	CVA/Stoke	Hypertension, Family History of CAD
VUHD87	Healthy Donor	53/Male	1p/d/7 years	Cardiac Arrest	Etoh Abuse, Hypertension, Family history of CAD
VUHD92	Healthy Donor	55/Male	unknown	CVA/Stroke	Hypertension, Family history of CAD
VUHD94	Healthy Donor	62/Male	unknown	Stroke	No medical history
VUHD140	Healthy Donor	61/Male	unknown	CVA	No medical history
VUILD72	IPF	62/Female	unknown		HTN,DM2, Dyslipidemia, GERD, Osteoarthritis, Rheumatoid Arthritis, Restless Leg Syndrome, Moderate Asthma
VUILD74	IPF	62/Male	10 PY quit 40 years ago		
VUILD76	IPF	53/Male	unknown		Obesity, Hyperlipidema
VUILD77	IPF	62/Male	30 PY quit 18 years ago		DM2, HTN, Hypothyroidism
VUILD79	IPF	59/Female	Quit 25 years ago		GERD, HLN
VUILD80	IPF	69/Female	unknown		No medical history
